# Deep-Sea-Derived Isobisvertinol Targets TLR4 to Exhibit Neuroprotective Activity via Anti-Inflammatory and Ferroptosis-Inhibitory Effects

**DOI:** 10.3390/md23010049

**Published:** 2025-01-20

**Authors:** Zi-Han Xu, Ming-Min Xie, Chun-Lan Xie, Xian-Wen Yang, Jun-Song Wang

**Affiliations:** 1 Center for Molecular Metabolism, School of Environmental and Biological Engineering, Nanjing University of Science and Technology, 200 Xiaolingwei Street, Nanjing 210094, China; xuzihan@njust.edu.cn; 2 School of Basic Medicine and Life Science, Hainan Academy of Medical Sciences, Hainan Medical University, 3 Xueyuan Road, Haikou 571199, China; xiechunlanxx@muhn.edu.cn; 3 Key Laboratory of Marine Genetic Resources, Third Institute of Oceanography, Ministry of Natural Resources, 184 Daxue Road, Xiamen 361005, China; 15717083707@163.com

**Keywords:** marine natural products, metabolomics, neuroprotection, anti-inflammatory, ferroptosis inhibition

## Abstract

Neuroinflammation and neuronal cell death are leading causes of death in the elderly and underlie various neurodegenerative diseases. These diseases involve complex pathophysiological mechanisms, including inflammatory responses, oxidative stress, and ferroptosis. Compounds derived from deep-sea fungi exhibit low toxicity and potent neuroprotective effects, offering a promising source for drug development. In this study, we isolated 44 natural products from deep-sea-derived fungi and identified isobisvertinol (**17**) as a compound with anti-inflammatory and ferroptosis-inhibiting effects. Using LPS-induced microglial inflammation and RSL3-induced neuronal ferroptosis models, we found that **17** targets TLR4 to provide neuroprotection. Molecular docking studies revealed that **17** inhibits TLR4 activation by occupying the hydrophobic pocket at the TLR4-MD2 binding site. Additionally, **17** suppresses TLR4, reducing p38 MAPK phosphorylation, and inhibits ferroptosis by decreasing lipid peroxidation and modulating mitochondrial membrane potential. Metabolomic analysis showed that **17** rescues alterations in multiple metabolic pathways induced by RSL3 and increases levels of antioxidant metabolites, including glutamine, glutamate, and glutathione. In summary, our results indicate that isobisvertinol (**17**) targets TLR4 in neural cells to reduce inflammation and inhibit p38 MAPK phosphorylation, while regulating metabolic pathways, mainly GSH synthesis, to provide antioxidant effects and prevent ferroptosis in neurons.

## 1. Introduction

Neurodegenerative diseases have become increasingly prevalent over the past three decades, affecting approximately 15% of the global population in some form [1]. Among them, Alzheimer’s disease, Parkinson’s disease, amyotrophic lateral sclerosis, and Huntington’s disease are the most common, posing significant challenges to global public health. The pathophysiological mechanisms of these diseases are complex, involving various factors such as inflammatory responses, oxidative stress, and ferroptosis, which interact with each other to contribute to neuronal damage and death [2,3]. The overproduction of nitric oxide (NO) and the occurrence of ferroptosis are both associated with an increase in reactive oxygen species (ROS), leading to oxidative stress when ROS levels surpass the cell’s antioxidant defenses [4,5]. These processes play a critical role in the progression of neurodegenerative diseases. Upon activation, microglia express inducible nitric oxide synthase (iNOS), producing large amounts of NO. NO reacts with superoxide anions (O_2_^−^) to form peroxynitrite (ONOO^−^), a potent ROS that can trigger neuroinflammation and further exacerbate neuronal damage [6,7]. Additionally, the accumulation of iron and the initiation of ferroptosis may lead to neuronal loss and dysfunction. Excessive iron accumulation enhances the Fenton reaction, thereby generating more ROS and intensifying oxidative stress [8]. Consequently, there is a pressing need to develop specific drugs targeting oxidative stress, inflammatory responses, and ferroptosis, aiming to reduce neuronal damage and death, and thus treat neurodegenerative diseases.

Toll-like receptor 4 (TLR4) is a pattern recognition receptor capable of identifying pathogen-associated molecular patterns (PAMPs), particularly the lipopolysaccharide (LPS) from Gram-negative bacteria [9]. Upon binding with LPS, TLR4 activates downstream signaling pathways, such as the nuclear factor-kappaB (NF-κB) pathway, leading to the production and release of pro-inflammatory cytokines and the initiation of inflammatory responses [10]. The activation of TLR4 can enhance inflammation-related oxidative stress, also mediated through the activation of transcription factors like NF-κB [11]. Additionally, TLR4 is associated with the activation of NADPH oxidase, which generates a significant amount of ROS, further exacerbating oxidative stress [12]. It is important to note that elevated intracellular ROS levels are a key driver of ferroptosis [8]. Studies have shown that TLR4 can promote ferroptosis by increasing ROS generation and regulating intracellular iron levels [13,14]. Currently, several small molecule drugs targeting TLR4 are in clinical trial stages, demonstrating potential therapeutic value in the fields of inflammatory diseases, cancer, and autoimmune diseases [15,16].

In recent years, marine natural products have shown great potential in the treatment of neurodegenerative diseases, such as Alzheimer’s and Parkinson’s diseases, due to their unique chemical structures and broad biological activities [17,18]. These compounds not only exhibit favorable pharmacological effects but also typically possess lower toxicity and better bioavailability, making them important candidates for the development of new drugs [19,20]. The uniqueness of the deep-sea environment has given rise to a plethora of structurally novel compounds that may serve as a new source of drugs for the treatment of neurodegenerative diseases.

**Figure 1:**
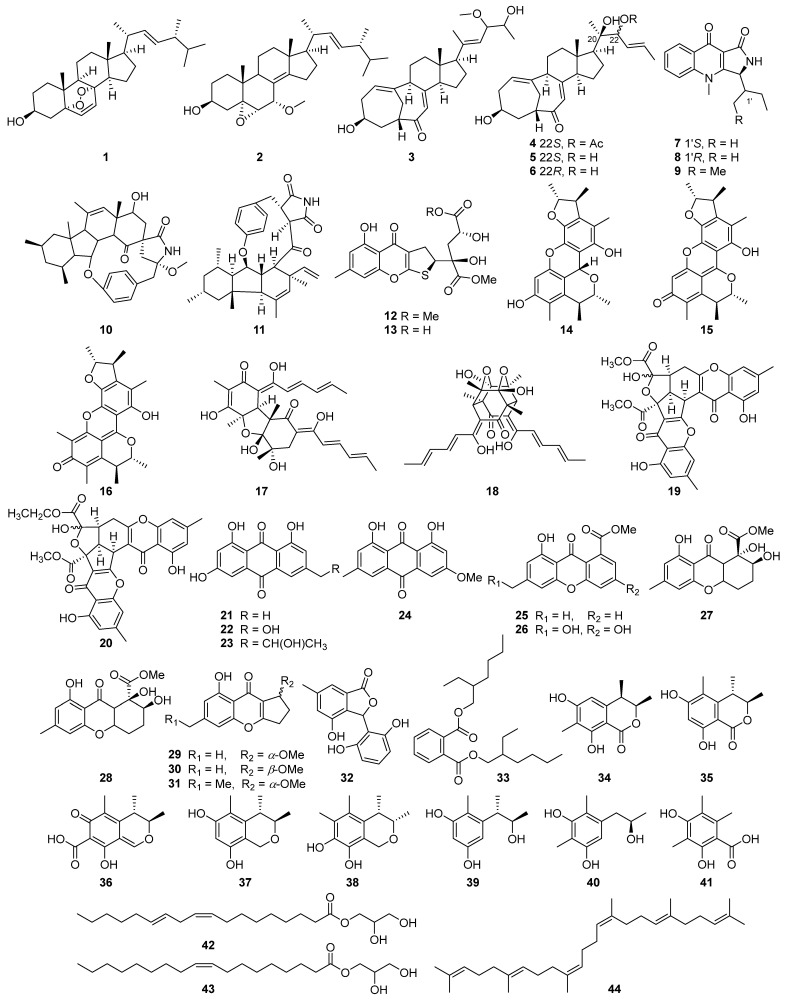
The chemical structures of compounds **1**–**44** isolated from *Penicillium griseofulvum*.

Previously, we found that the fermentation extract of *Penicillium griseofulvum* exhibited significant anti-inflammatory activity (Figure S1). Therefore, a systematic chemical investigation was performed on this strain (Scheme S1). As a result, 44 known compounds were obtained (Figure 1). Given the known anti-inflammatory activity of sorbicilinoid-type compounds [21,22], isobisvertinol (**17**) was subjected to anti-inflammatory and ferroptosis-inhibitory studies using network pharmacology, metabolomics, and pathway analysis approaches to elucidate the mechanisms of action from a metabolic perspective. Herein, we report on the structures and bioactivities of all isolated compounds from *Penicillium griseofulvum*.

## 2. Results

### 2.1. Chemical Constituents of Penicillium griseofulvum

By comparison of NMR, MS and optical rotation data with those reported in the literature, 44 previously described compounds were identified as 22*E*-5*α*,8*α*-epidioxyergosta-6,9(11),22-trien-3*β*-ol (**1**) [23], 22*E*-7*α*-methoxy-5*α*,6*α*-epoxyergosta-8(14),22-dien-3*β*-ol (**2**) [24], *threo*-23-*O*-methylneocyclocitrinol (**3**) [25], 22-acetylisocyclocitrinol A (**4**) [26], isocyclocitrinol A (**5**) [25], isocyclocitrinol B (**6**) [25], quinolactacin A1 (**7**) [27], quinolactacin A2 (**8**) [27], quinolactacin-H (**9**) [28], pyrrospirones C (**10**) [29], GKK1032B (**11**) [30], 11-*O*-methyl-oxalicumone A (**12**) [31], oxalicumone A (**13**) [31], penicitrinol A (**14**) [32], penicitrinone A (**15**) [32], penicitrinone F (**16**) [33], isobisvertinol (**17**) [34], trichodimerol (**18**) [35], epiremisporine B (**19**) [36], epiremisporine B1 (**20**) [36], emodin (**21**) [37], citreorosein (**22**) [38], isorhodoptilometrin (**23**) [39], physcion (**24**) [40], janthinone (**25**) [41], conioxanthone A (**26**) [42], *α*-diversonolic ester (**27**) [43], *β*-diversonolic ester (**28**) [43], coniochaetone J (**29**) [44], (−)-coniochaetone E (**30**) [45], (+)-coniochaetone E (**31**) [45], 3-(2,6-dihydroxyphenyl)-4-hydroxy-6-methyl-isobenzofuran-1(3H)-one (**32**) [46], bis(2-ethylhexyl)benzene-1,2-dicarboxylate (**33**) [47], sclerotinin C (**34**) [48], stoloniferol B (**35**) [49], citrinin (**36**) [50], decarboxydihydrocitrinin (**37**) [32], guhypoxylonol C (**38**) [51], phenol A (**39**) [52], 5-[2*R*-2-hydroypropane-1-yl]-2,6-dimethlbenzene-1,3-diol (**40**) [53], 3,5-dimethylorsellinic acid (**41**) [54], 1-glyceryl linoleate (**42**) [55], glycerol-1-monooleate (**43**) [56], and squalene (**44**) [57].

### 2.2. Screening of Compounds for the Ability to Inhibit LPS-Induced Microglial Neuroinflammation

**Figure 2:**
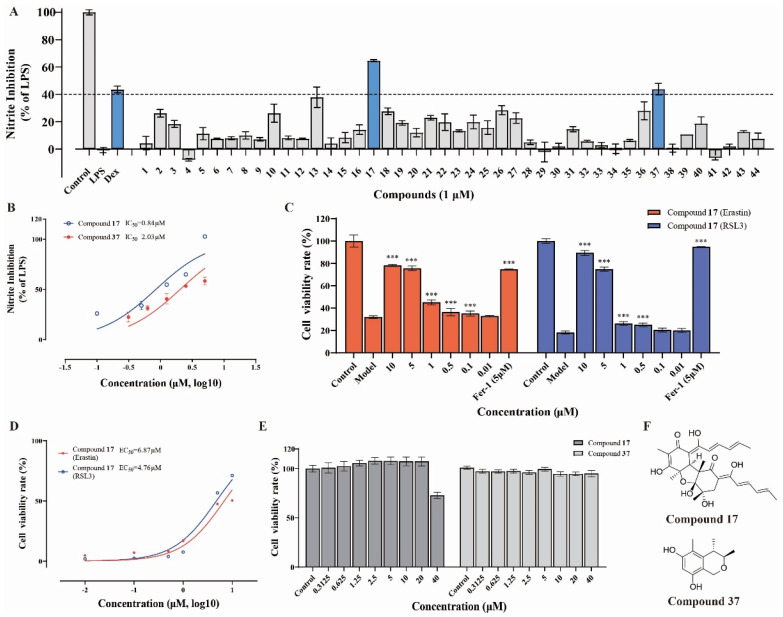
Identification of neuroprotective compounds through their anti-inflammatory and ferroptosis-inhibitory properties. (**A**) Evaluation of the anti-inflammatory potential of marine-derived compounds **1**–**44** in BV2 cells, highlighting the inhibitory effects on nitric oxide (NO) production. (**B**) Determination of IC_50_ values for isobisvertinol (**17**) and decarboxydihydrocitrinin (**37**), demonstrating their potency in inhibiting NO production, with **17** showing an IC_50_ of 0.84 μM and **37** an IC_50_ of 2.03 μM. (**C**) Visualization of the protective effects of **17** against ferroptosis triggered by Erastin and RSL3, comparing its efficacy to that of the positive control, Fer-1. (**D**) Assessment of the EC_50_ values for **17** in protecting against ferroptosis induced by RSL3 and Erastin, with EC_50_ values of 4.76 μM and 6.87 μM, respectively; (**E**) Analysis of the cytotoxicity profile of **17** and **37** at various concentrations, indicating no significant cytotoxicity within the tested range. (**F**) Presentation of the chemical structures of the compounds **17** and **37**, which were identified as promising candidates for their neuroprotective properties. Data were expressed as mean ± SEM. *** *p* < 0.001 compared with the Model group.

Nitric oxide (NO) is widely recognized as a significant factor in inflammatory processes. To investigate the anti-inflammatory effects of compounds **1**–**44** in neuronal cells, we conducted an NO production inhibition assay in LPS-induced BV-2 cells. As shown in Figure 2A,B, compounds isobisvertinol (**17**) and decarboxydihydrocitrinin (**37**) both exhibited good inhibitory effects at a concentration of 1 μM (superior to the positive control drug Dex), with IC_50_ values of 0.84 and 2.03 μM, respectively. Furthermore, in the CCK8 assay, **17** and **37** did not exhibit cytotoxicity at the concentrations tested in this study (Figure 2E).

### 2.3. Isobisvertinol (**17**) Prevented RSL3 or Erastin-Induced Ferroptosis in HT-22 Cells

According to the existing literature, ferroptosis has been associated with neuroinflammation. Therefore, we aimed to explore the potential of isobisvertinol (**17**) in the amelioration of ferroptosis. In this study, experiments were performed using the HT-22 cell line, with the compound being introduced and incubated for 1 h before the cells were treated with 2.5 μM RSL3 for 12 h or 10 μM Erastin for 24 h to trigger ferroptosis, consistent with previous methodologies. Moreover, 10 μM Fer-1, recognized as a potent inhibitor of ferroptosis, served as the positive control. Our findings revealed that **17** could effectively alleviate ferroptosis induced by different inducers, with an efficacy similar to that of the positive control, and showed enhanced potency in the presence of Erastin (Figure 2C). The EC_50_ values were 4.76 μM for RSL3 and 6.87 μM for Erastin, respectively (Figure 2D).

### 2.4. Functional Enrichment Analysis

**Figure 3:**
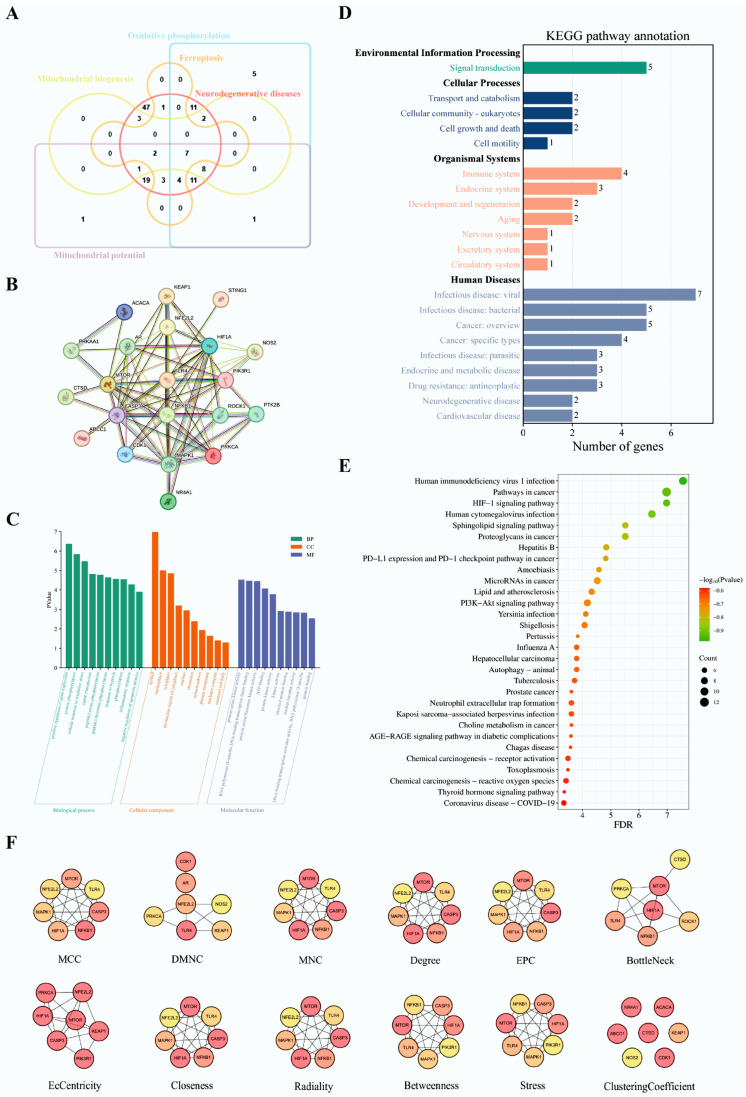
Functional enrichment. (**A**) Multivariate Venn diagram of isobisvertinol-disease/biofunction targets. (**B**) PPI network of targets with more than three intersections in the multivariate Venn diagram. (**C**) Functional enrichment cluster analysis of the top 10 GO terms in Biological Process (BP), Cellular Component (CC), and Molecular Function (MF). (**D**) Secondary classification annotation of KEGG. (**E**) Bubble chart of the top 30 enriched pathways. (**F**) CytoHubba algorithm results. Analysis of the PPI network data from STRING using Cytoscape, with hub gene identification performed using 12 algorithms included in cytoHubba, ultimately selecting the top 7 genes ranked by each algorithm’s scoring system as hub genes.

In order to predict the pharmacological mechanism of isobisvertinol (**17**) in the treatment of inflammatory and ferroptosis, network pharmacology analysis was conducted. Functional enrichment clustering analysis revealed a significant association between intersected genes and the inflammatory/ferroptosis (Figure 3A). We selected the top 10 gene ontology (GO) terms in each of the biological processes, cellular component, and molecular function (Figure 3C). Cellular response to oxidative stress, inflammatory response and negative regulation of apoptotic process were related GO terms identified in our analysis. In the Kyoto encyclopedia of genes and genomes (KEGG) pathway annotation (Figure 3D), we found that pathways associated with immune, nervous system, cancer, and neurodegenerative diseases are associated with inflammatory and ferroptosis. The top 30 pathway enrichment analysis is shown in Figure 3E, where bubble size represents gene number and color represented *p* value.

### 2.5. Hub Gene Identification and Predicting TLR4 as a Key Target

The protein-protein interaction (PPI) network analysis revealed a complex structure comprising 21 nodes and 80 edges (Figure 3B). Further analysis of STRING results using Cytoscape, specifically through the cytoHubba algorithms, identified key hub genes (Figure 3F). Seven hub genes were identified based on their frequency of appearance across different algorithms: TLR4 (11), CASP3 (10), HIF1A (10), MAPK1 (10), MTOR (10), NF-κB 1 (10), and NFE2L2 (NRF2) (8), with the numbers in parentheses indicating their cumulative algorithmic occurrence.

**Figure 4:**
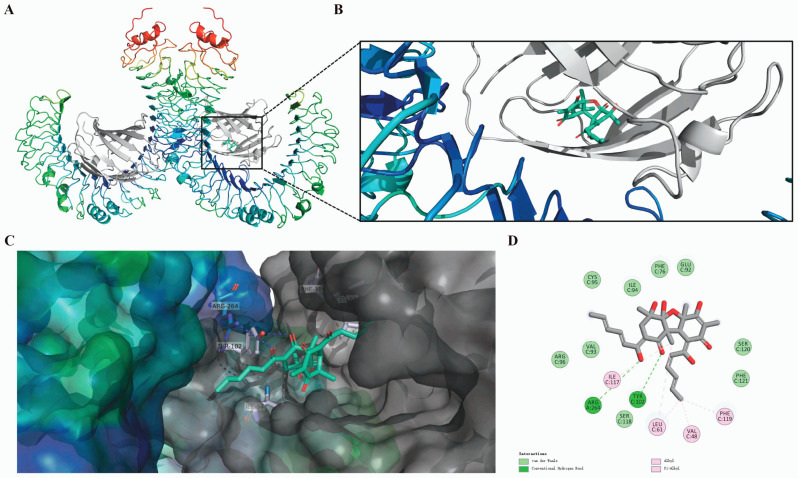
Molecular docking configuration of isobisvertinol (**17**) to the toll-like receptor 4 (TLR4)/myeloid differentiation protein-2 (MD-2) complex. (**A**) Molecular docking results of **17** and TLR4/MD-2 complex, the total view. (**B**,**C**) Side view of symmetrical dimer of the complex and **17** binding site in the crystal structure. (**D**) The detailed interaction view of TLR4/MD-2 pertinent amino acids residues with **17**.

Based on cytoHubba analysis, TLR4, which achieved the highest score, was selected for molecular docking studies with isobisvertinol (**17**). Given that myeloid differentiation protein-2 (MD-2) functions as an essential accessory molecule for TLR4 in LPS recognition, we investigated the interaction between **17** and the TLR4/MD-2 complex (PDB: 3FXI). The optimal binding configuration was identified, characterized by high binding affinity and a favorable binding free energy of −8.1 kcal/mol (Figure 4A,B).

Analysis of potential binding sites (Figure 4C) revealed that **17** occupied a hydrophobic pocket formed by specific amino acid residues, including arginine (Arg) 264, tyrosine (Tyr) 102, leucine (Leu) 61, phenylalanine (Phe) 119, and valine (Val) 48 (Figure 4D). The molecular docking analysis demonstrated multiple interaction types between **17** and the TLR4/MD-2 complex, with hydrogen bonding being predominant. Specifically, two hydrogen bonds were formed between the keto and hydroxyl groups of **17** and the amino acid residues Arg 264 and Tyr 102 of the TLR4/MD-2 protein (Figure 4D). These hydrogen bonds exhibited strong interactions, with donor–acceptor distances of 4.0 and 3.2 Å. Additionally, **17** established π-alkyl interactions with Val 48, Leu 61, and Phe 119, with distances ranging from 3.7 to 4.1 Å, while being stabilized within the hydrophobic cavity of the TLR4/MD2 protein through interactions with surrounding hydrophobic and hydrophilic residues.

### 2.6. Isobisvertinol (**17**) Inhibits Neuroinflammation by Down-Regulating TLR4 Protein Expression and p38 Phosphorylation

**Figure 5:**
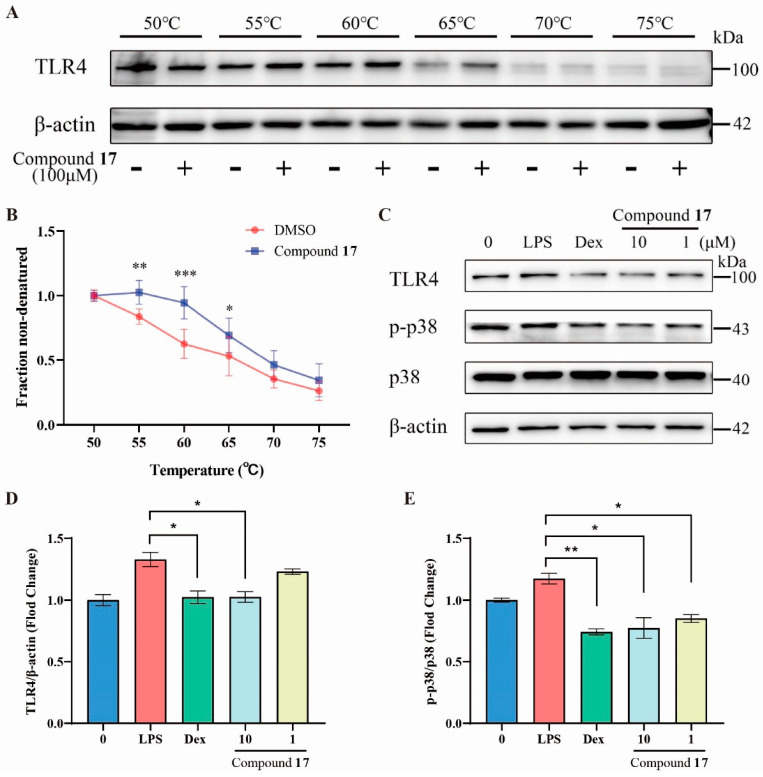
Validation of anti-inflammatory pathways for isobisvertinol (**17**). (**A**) Cellular thermal shift assay analysis of the binding between **17** and TLR4 protein. (**B**) Quantification of (**A**). (**C**) Western blot analysis of TLR4 and p-p38/p38 in BV2 cells. (**D**,**E**) Quantification of (**C**). Data were represented as mean ± SEM. * *p* < 0.05, ** *p* < 0.01, and *** *p* < 0.001 compared with the DMSO or LPS group.

Cellular thermal shift assay is a technique used to detect the binding efficiency of drugs to their target proteins. The principle is that when proteins bind to drug molecules, they typically become more stable, leading to an increase in the protein’s melting temperature value [58]. As shown in Figure 5A,B, cellular thermal shift assay (CETSA) results indicate that isobisvertinol (**17**) enhances TLR4 protein thermostability upon heating from 50 °C to 75 °C, implicating TLR4 as a key target for isobisvertinol’s anti-inflammatory activities. Figure 5C–E shows that **17**, at 10 μM and 1 μM, inhibits LPS-induced TLR4 upregulation and p38 mitogen-activated protein kinase (MAPK) phosphorylation, similar to Dex. These findings suggest the potential of **17** for modulating inflammatory responses through specific molecular interactions.

### 2.7. Isobisvertinol (**17**) Ameliorated RSL3-Induced Lipid Peroxidation Accumulation in HT-22 Cells

**Figure 6:**
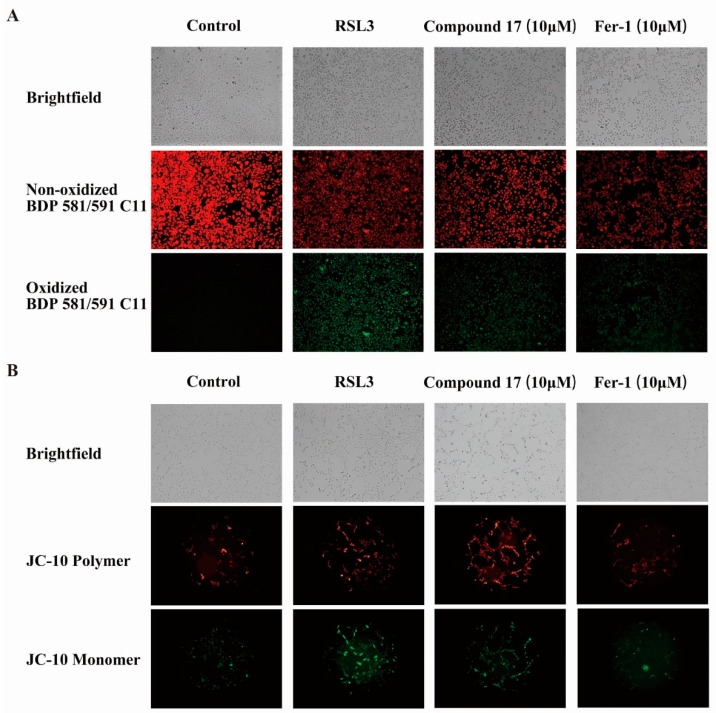
Inhibition of ferroptosis by isobisvertinol (**17**) through reducing lipid peroxide levels and modulating mitochondrial membrane potential. (**A**) Determination of lipid peroxidation levels with BODIPY581/591C11 after **17** treatment. (**B**) Measurement of mitochondrial membrane potential with JC-10 following **17** treatment.

Given that lipid peroxidation is a characteristic marker of ferroptosis, we employed 10 μM Fer-1 as a positive control and utilized BODIPY581/591C11 (BODIPYC11) staining to assess lipid peroxidation levels. The results demonstrated that RSL3 treatment led to significant accumulation of lipid peroxidation compared to the control group (Figure 6A). Notably, this RSL3-induced lipid peroxidation was substantially attenuated when cells were co-treated with either isobisvertinol (**17**) or Fer-1. These findings collectively indicate that **17** enhances cellular antioxidant capacity and effectively protects HT-22 cells against RSL3-induced ferroptosis.

### 2.8. Isobisvertinol (**17**) Ameliorated RSL3-Induced Collapse of Mitochondrial Membrane Potential in HT-22 Cells

Given that mitochondria are the primary cellular organelles for the production of ROS, we next evaluated mitochondrial function by analyzing the mitochondrial membrane potential (MMP) measured through JC-10 staining. As depicted in Figure 6B, isobisvertinol (**17**) significantly reduced the degree of MMP depolarization induced by RSL-3. Moreover, compared to the control group, the MMP levels in the **17**-treated group remained elevated. These findings indicate that **17** ameliorates mitochondrial dysfunction in RSL3-impaired cells.

### 2.9. Metabolomics Analysis

**Figure 7:**
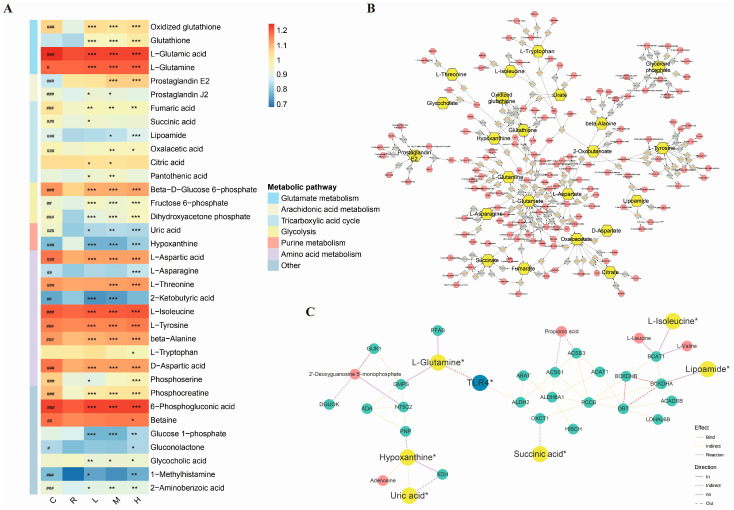
Metabolomic profiling to determine the protective influence of isobisvertinol (**17**) on ferroptosis. (**A**) Heatmap of median-scaled metabolite abundances generated via hierarchical clustering, distinguishing Control, RSL3, and **17** (1, 3, 10 μM) + RSL3 groups. Blue and red represent low and high relative metabolite levels, respectively. *p*-values corrected by Benjamani–Hochberg methods were calculated based on a parametric Student’s *t* test or a nonparametric Mann–Whitney test. PLS-DA showed separation between experimental groups. (**B**) Metabolite-reaction interaction network constructed to visualize connections between metabolites and reaction. Yellow nodes represent the differential metabolites detected. Red nodes represent relevant metabolites involved in metabolic reactions. Celadon nodes represent metabolic reactions. (**C**) Target-metabolite-gene interaction network constructed to visualize connections between genes and metabolites. Blue nodes represent target genes. Green nodes indicate genes potentially involved in metabolism. Red and yellow nodes correspond to those in (**B**). * *p* < 0.05, ** *p* < 0.01, *** *p* < 0.001 compared with **17** treatment and RSL3 group. # *p* < 0.05, ## *p* < 0.01, ### *p* < 0.001 compared with the control and RSL3 group.

To elucidate the metabolic alterations in HT22 cells following treatment with isobisvertinol (**17**) and RSL3, UHPLC-Q/TOF MS data were subjected to partial least-squares discriminant analysis (PLS-DA) analysis. Metabolites with a VIP value greater than 1 obtained from PLS-DA were selected to identify significant differences among groups. As shown in Figure 7A, the heatmap displays differential metabolites between the control and RSL3 groups, indicating the successful induction of a ferroptosis injury model and changes in the metabolic profile. Most differential metabolites in the **17** + RSL3 groups were similar to those in the RSL3 group, with a notable increase in glutathione levels post-treatment, suggesting a protective effect of **17** against ferroptosis. Incorporating the identified differential metabolites into the KEGG metabolic network yielded a metabolite-reaction network (Figure 7B), indicating that **17** exerts its anti-ferroptotic effects by regulating multiple interconnected metabolic pathway nodes. Furthermore, as depicted in Figure 7C, mapping the target TLR4 onto the metabolic network resulted in a target–metabolite–gene association network. This network highlights the metabolites associated with the target and the genes involved in the regulatory process. Collectively, these results strongly suggest that **17** protects neurons from RSL3-induced ferroptosis by modulating these key signaling cascades.

## 3. Discussion

In neurodegenerative diseases, the accumulation of excessive ROS leads to cellular damage and death. The overproduction of the inflammatory factor NO can induce oxidative stress, and ferroptosis is characterized by an increase in lipid peroxidation, both of which are closely related to the accumulation of ROS [59,60]. Under normal conditions, cells rely on antioxidants such as glutathione to combat ROS-induced damage. Our study found that isobisvertinol (**17**) significantly inhibited the generation of NO in LPS-induced neuroinflammation models and suppressed ferroptosis in neuronal cells, similar to previously reported anti-inflammatory and antioxidant activities of marine natural products [19,61]. This suggests that **17** possesses potential anti-inflammatory and neuroprotective effects.

Through network pharmacology analysis and molecular docking methods, we predicted the pharmacological mechanisms of **17** in treating inflammation and ferroptosis. The PPI network of intersecting genes revealed that TLR4, caspase-3 (CASP3), hypoxia inducible factor 1 alpha (HIF1A), MAPK1, mechanistic target of rapamycin (MTOR), and NF-κB1 are potential key genes involved in the regulation of neural function by this compound. Hub gene identification algorithms further predicted TLR4 as a potential target. Subsequent molecular docking and CETSA results also confirmed the possibility of TLR4 as a target for **17** in modulating inflammation and ferroptosis (Figure 4 and Figure 5). Interestingly, the structural backbone of **17** is similar to disaccharide lipid A mimetics (DLAMs). DLAMs tightly bind to the hydrophobic pocket between TLR4 and MD-2 through their coplanar disaccharide backbone and fatty acid chain [62]. Similarly, **17** likely prevents the binding of MD-2 to TLR4 via LPS, thereby inhibiting TLR4 activation (Figure 4). Functional enrichment cluster analysis revealed a significant association between intersecting genes and inflammation/ferroptosis, including terms such as ‘cellular response to oxidative stress’, ‘signal transduction’, ‘inflammatory response’, and ‘mitochondrion’, suggesting that TLR4 may exert anti-inflammatory and antioxidant effects through cellular signal transduction. This is consistent with the expression patterns of genes related to inflammation and ferroptosis reported in previous studies [59]. In the KEGG pathway secondary annotation, most enriched genes belong to pathways related to immunity, the nervous system, and neurodegenerative diseases. The ranking of the top 30 enriched pathways also showed that pathways related to inflammation and ferroptosis, such as the HIF-1 signaling pathway, Hepatitis B, lipid and atherosclerosis, and PI3K-Akt signaling pathway, were ranked at the forefront. Therefore, **17** may inhibit inflammation and ferroptosis through the mediation of multiple key targets such as TLR4 and multiple signaling pathways, including the PI3K-Akt signaling pathway.

TLR4 has been proven to be closely related to the initiation of inflammatory responses which is an important receptor protein for LPS-induced inflammation [9]. Our results (Figure 5A,B) show that **17** increased the stability of TLR4 protein in the samples, suggesting that **17** may interact with TLR4, thereby exerting its anti-inflammatory and ferroptosis-inhibiting effects. p38 MAPK can be activated by various stress stimuli, including LPS, chemokines, and ultraviolet radiation [63]. Studies have shown that the TLR4 inhibitor TAK-242 exerts neuroprotective and anti-ferroptotic effects by inhibiting TLR4-p38 MAPK signaling, reducing the expression of ferroptosis-related genes and pro-inflammatory factors [64]. Similarly, we found that **17** could inhibit the upregulation of TLR4 protein levels induced by LPS and suppress the occurrence of inflammation by inhibiting p38 phosphorylation (Figure 5C). TLR4 not only promotes ferroptosis by up-regulating intracellular iron levels and the accumulation of lipid peroxides (L-ROS) but also enhances ferroptosis by downregulating the expression of GPX4 [64,65]. Another study showed that in a viral myocarditis model, TLR4 overexpression enhanced ferroptosis, while TLR4 inhibition alleviated it [66]. Numerous studies have indicated that mitochondria play a key role in the process of ferroptosis [67,68]. Mitochondria are not only the main site of lipid peroxidation but also affect ferroptosis by regulating intracellular iron ion levels and the activity of antioxidant systems such as GPX4. Our network pharmacology GO enrichment CC results also suggest that **17** may exert its effects by influencing mitochondrial function (Figure 3C). Consistent with the prediction in our neuronal ferroptosis model, **17** not only inhibits ferroptosis by reducing the accumulation of L-ROS but also improves the mitochondrial membrane potential disorder caused by ferroptosis inducers (Figure 6). Therefore, these results collectively suggest that **17** modulates neuroinflammation and ferroptosis by targeting the TLR4-p38 MAPK signaling pathway, thereby exerting neuroprotective effects.

To further elucidate the underlying molecular mechanisms, we employed a UHPLC-QTOF-MS-based metabolomics approach to identify metabolic changes in HT22 cells following treatment with **17** and RSL3. The metabolic heatmap and reaction network highlighted metabolic reaction networks primarily involving glutathione synthesis (glutamate metabolism), the citric acid cycle, and amino acid metabolism. The target–metabolite reaction network revealed that TLR4 mediates glutathione synthesis through glutamine and indirectly regulates multiple metabolic pathways, including purine metabolism, the citric acid cycle, and amino acid metabolism.

Glutamine is one of the essential amino acids in cells, participating in various metabolic pathways, including the synthesis of glutathione (GSH) [69]. GSH is a crucial antioxidant that protects cells from oxidative stress damage [70]. Studies have shown that the increased metabolism of glutamine can elevate GSH levels, thereby enhancing the cell’s antioxidant capacity [69]. GSH can directly react with ROS, reducing them to stable compounds [71,72]. Moreover, GSH is a substrate for glutathione peroxidase (GPX), which catalyzes the reduction in L-ROS to their corresponding alcohols, thus protecting cells from lipid peroxidation damage [73]. Previous research has indicated that **17** can reduce the aggregation of lipid droplets [34]. Our study further reveals that this effect may be closely associated with the increase in GSH levels (Figure 7). During ferroptosis, the depletion of GSH and the decline in GPX4 activity lead to a reduced antioxidant capacity in cells, causing lipid peroxidation and metabolic dysfunction, with an increase in L-ROS, which are the main causes of ferroptosis [67,68,73]. Therefore, **17** can effectively inhibit ferroptosis by increasing the synthesis of GSH.

While there is no direct evidence to suggest that inhibiting TLR4 can directly enhance glutamine metabolism, the activation of TLR4 typically leads to inflammatory responses and oxidative stress, processes that deplete GSH levels [10,11,12]. Metabolomic heatmaps (Figure 7A) reveal that **17** can significantly increase the content of glutamine, glutamic acid (glutamate), GSH, and oxidized glutathione (GSSG) in neural cells. Therefore, we propose that under conditions of inflammation or ferroptosis in neural cells, inhibiting TLR4 might promote glutamine metabolism, thereby increasing GSH levels and ultimately reducing oxidative stress and inflammation. Notably, several studies have shown that glutamine can alleviate inflammatory responses by inhibiting the TLR4 signaling pathway [74,75,76]. For instance, in intestinal inflammation models, glutamine supplementation reduces TLR4 expression, thereby mitigating inflammation [76]. Our results also suggest this effect (Figure 5C). This indicates a possible negative feedback regulation between TLR4 and glutamine.

Beyond GSH synthesis, metabolomic analysis revealed that treatment of neuronal cells with RSL3 led to a reduction in the synthesis of various metabolites, including hypoxanthine, uric acid, succinate, lipoamide, and L-isoleucine, among others. However, treatment with **17** rescued these changes. Metabolic alterations in glutamate, hypoxanthine, and uric acid are involved in the purine metabolism pathway (Figure 7A–C), and these changes can impact cellular energy metabolism and antioxidant capacity. The tricarboxylic acid (TCA) cycle is a primary pathway for cellular energy metabolism, and its perturbations may affect cellular energy supply and metabolic balance [77]. Intermediates of the TCA cycle, such as succinate, lipoamide, and fumarate, were also affected by RSL3 and **17** (Figure 7A). Ferroptosis leads to a reduction in certain key metabolites of the TCA cycle [67], due to ROS generated during ferroptosis damaging mitochondrial function and affecting the normal progression of the TCA cycle. Compound **17** was able to ameliorate the negative impact of ferroptosis on the TCA cycle. By restoring the TCA cycle, 17 may indirectly reduce oxidative stress and inflammatory responses induced by ferroptosis, which are crucial for the survival and function of neural cells. Changes in the levels of L-isoleucine, L-leucine, and L-valine, involved in branched-chain amino acid metabolism, were also significantly altered under RSL3 and **17** treatment (Figure 7A), and these amino acids play important roles in neuroprotection and cellular stress responses [78]. Notably, our results suggest that TLR4 may indirectly regulate isoleucine through multiple genes such as aldehyde dehydrogenase 2 (ALDH2)-BCAA transaminase 1 (BCAT1) (Figure 7C), which plays a role in energy metabolism, participating in gluconeogenesis and fatty acid synthesis [79,80,81]. Isoleucine is also involved in the regulation of the MTOR pathway (one of the predicted proteins in Figure 2), which is essential for cell growth and division [82]. Furthermore, **17** may modulate intermediates of the TCA cycle by affecting glycolysis and the pentose phosphate pathway, thereby helping to balance the redox status within cells and reducing lipid peroxidation induced by ferroptosis (Figure 7A,B). Our metabolomic study shows that **17** influences ferroptosis through multiple pathways, including GSH synthesis, purine metabolism, the TCA cycle, and amino acid metabolism.

**Figure 8:**
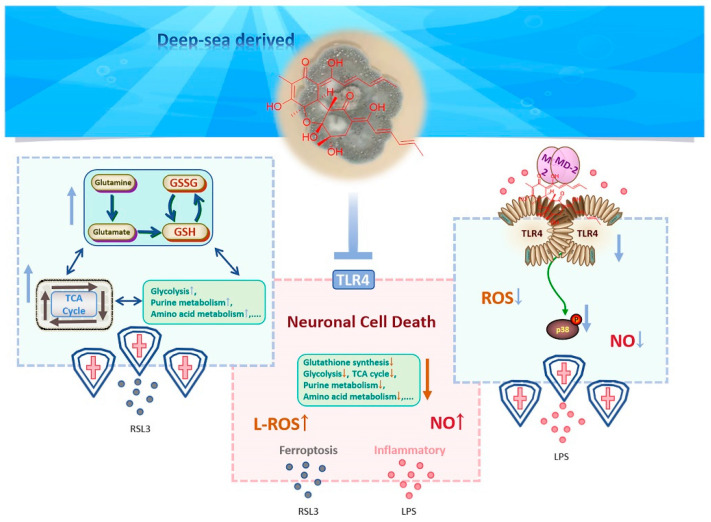
The schematic diagram of the potential mechanism of isobisvertinol (**17**).

In summary, our study demonstrates that isobisvertinol (**17**) exerts its anti-inflammatory effects by targeting TLR4 in neural cells and inhibiting the phosphorylation of p38 MAPK. Additionally, it modulates multiple metabolic pathways, primarily involving GSH synthesis, to exert antioxidant effects and inhibit ferroptosis in neurons. These findings help to elucidate the molecular mechanisms of isobisvertinol’s anti-inflammatory and ferroptosis-inhibiting actions, suggesting its potential utility in providing neuroprotection and treating neurodegenerative diseases (Figure 8).

## 4. Materials and Methods

### 4.1. General Experimental Procedures

NMR spectra were recorded on a Bruker 400 MHz spectrometer. The HRESIMS spectra were recorded on a Xevo G2 Q-TOF mass spectrometer (Waters Corporation, Milford, MA, USA). Optical rotations were obtained with an Anton Par polarimeter (MCP100). Preparative and semipreparative HPLC were performed on an Agilent Technologies 1260 infinity instrument equipped with the DAD detector. UV spectra were recorded on a UV-8000 UV/Vis spectrometer (Shanghai Yuanxi Instrument Co, Ltd., Shanghai, China). Column chromatography (CC) was performed on ODS (50 µm, Daiso, Hiroshima, Japan), silica gel (Qingdao Marine Chemistry Co., Ltd., Qingdao, China), and Sephadex LH-20 (Amersham Pharmacia Biotech AB, Uppsala, Sweden). The TLC plates were visualized under UV light or by spraying with 10% H_2_SO_4_. Solvents for isolation were analytical grade.

### 4.2. Fungal Identification, Fermentation, and Extraction

The fungus strain was isolated from the deep-sea sediment sample of the Southwest Pacific Ocean (−2484 m, W 176.31°, S 21.533°), and identified as *Penicillium griseofulvum* according to the sequencing of the ITS region (GenBank accession number EU497956). The specimen of the fungus was preserved in the Marine Culture Collection of China with the accession number of MCCC 3A00181.

The fungus was cultured on a PDA plate at 25 °C for 3 days to obtain fresh mycelia and spores. They were then inoculated in 3 × 1 L Erlenmeyer flasks containing 400 mL of PDB medium to obtain seed medium after culturing in a rotary shaker at 180 rpm under 28 °C for 2 days. Finally, 10 mL seed cultures were transferred into a total of 100 flasks (1 L) containing rice solid medium (100.0 g rice, 1.5 g sea salt, 120 mL water). The flasks were cultured statically at 25 °C for 28 days. The fermentation product was extracted by ethyl acetate (EtOAc) three times to yield a crude extract (100.0 g).

### 4.3. Isolation and Purification

The crude extract was separated into four fractions (Fr.1−Fr.4) via medium-pressure liquid chromatography (MPLC) on silica gel, with a gradient of CH_2_Cl_2_-MeOH (100%→70%). Fraction Fr. 1 (19.0 g) was subjected to column chromatography (CC) over ODS with MeOH-H_2_O (40%→80%), followed by purification using CC on Sephadex LH-20 (MeOH) to give **2** (4.4 mg), **36** (30.0 mg), **37** (5.2 mg), and **38** (3.3 mg). Fraction Fr.2 (25.0 g) was CC over ODS with MeOH-H_2_O (30%→70%), followed by purification using recrystallization or CC on Sephadex LH-20 and silica gel to provide **4** (3.4 mg), **5** (26.1 mg), **6** (1.1 mg), **7** (1.2 mg), **8** (1.0 mg), **9** (39.0 mg), **21** (5.2 mg), **22** (2.2 mg), **23** (10.0 mg), **24** (2.2 mg), **25** (10.0 mg), **26** (2.2 mg), **27** (10.0 mg), **28** (14.0 mg), **29** (22.0 mg), **30** (5.0 mg), **31** (2.2 mg), **32** (7.1 mg), and **33** (8.4 mg). Fraction Fr.3 (4.6 g) was separated by CC over ODS with MeOH-H_2_O (20%→60%) and Sephadex LH-20 (MeOH), followed by semi-prep. HPLC to afford **1** (9.0 mg), **3** (152.0 mg), **10** (1.7 mg), **11** (11.0 mg), **12** (3.3 mg), **13** (1.5 mg), **14** (8.2 mg), **15** (39.0 mg), **16** (1.2 mg), **17** (8.7 mg), **18** (6.7 mg), **19** (3.0 mg), **20** (10.0 mg), **42** (174.0 mg), and **43** (4.0 mg). Using similar procedures, **34** (90.0 mg), **35** (11.0 mg), **39** (13.2 mg), **40** (14.6 mg), **41** (3.0 mg), and **44** (2.2 mg) were obtained from fraction Fr.4 (5.0 g).

### 4.4. Materials

Dulbecco’s Modified Eagle Medium (DMEM), trypsin, and antibiotics were obtained from Basal Media Technologies (Shanghai, China, L120KJ). Fetal bovine serum and Griess Reagent Kit were obtained from Thermo Fisher Scientific, Inc. (Shanghai, China, A5669701). Lipopolysaccharide (LPS) was obtained from Sigma-Aldrich, Inc. (Shanghai, China, L4391). Dexamethasone (D129705), Ferrostatin-1 (F129882), Erastin (E126853) and RSL3 (R302648) were supplied by Aladdin Biochemical Technology Co., Ltd. (Shanghai, China). The Cell Counting Kit-8 (CCK8) was purchased from Topscience Biotechnology Co., Ltd. (Shanghai, China, C0005). The levels of mitochondrial membrane potential were measured using commercial kits (JC-10) from LABLEAD Co., Ltd. (Beijing, China, J2204). BODIPY581/591C11 was supplied by Dojindo Chemical Technology Co., Ltd. (Dojindo, Shanghai, L267). RIPA Lysis Buffer was from Servicebio Co., Ltd. (Wuhan, China, G2002). Antibodies against TLR4 (66350-1-Ig) and *β*-actin (60008-1-Ig) were obtained from Proteintech (Wuhan, China), and the antibody against p38 (#8690S) and p-p38 MAPK (#4511S) were acquired from Cell Signaling Technology (Danvers, MA, USA). The enhanced chemiluminescent (ECL) plus reagent kit was obtained from Advansta Inc. (San Jose, CA, USA, K-12045-D50).

### 4.5. Cell Culture

The murine microglial cell line BV-2 was maintained by our laboratory, while the murine neuronal cell line HT22 was purchased from Wuhan Procell Life Science and Technology Co., Ltd. (Wuhan, China). Both cell lines were cultured in DMEM, supplemented with 10% FBS and 1% antibiotics, at 37 °C in a humidified incubator with 5% CO_2_. Cells were passed 30–40 times or replaced from the original frozen stock every 3 months.

### 4.6. Anti-Inflammatory Activity

BV-2 cell culture and compound treatment were performed as previously reported [83]. Briefly, BV-2 microglia cells were cultured in DMEM supplemented with 10% fetal bovine serum and 1% antibiotics at 37 °C in a humidified incubator with 5% CO_2_. Cells were seeded on a 24-well plate at a density of 1.5 × 10^5^ cells per well and allowed to adhere overnight. The following day, cells were treated with fresh medium containing the tested compounds at specified concentrations for 1 h, followed by exposure to LPS (1 µg/mL). Control groups were treated with a 0.1% DMSO solution. The concentration of nitrite present in the culture medium was assessed using the Griess Reagent Kit. Following the manufacturer’s instructions, the absorbance at a wavelength of 560 nm was recorded using the Multimode Mithras LB 943 microplate reader (Berthold, Germany).

### 4.7. Cell Viability Assay

HT22 cell culture and compound treatment were performed as previously reported [84]. Cells were cultured in DMEM supplemented with 10% fetal bovine serum and 1% antibiotics at 37 °C in a humidified incubator with 5% CO_2_ until they reached the logarithmic growth phase. HT22 cells were seeded into a 24-well plate at a density of 5 × 10^4^ cells per well and allowed to adhere overnight. The following day, cells were pre-treated with compounds of interest for 1 h, followed by treatment with RSL3 to induce ferroptosis. After 12 h of RSL3 treatment, cell viability was assessed using the CCK8 assay, and absorbance at 480 nm was recorded with a Multimode Mithras LB 943 microplate reader, following the manufacturer’s protocol.

### 4.8. Cytotoxicity Test

BV2 cells were seeded in 96-well plates at a density of 5 × 10^3^ cells per well with 200 μL of culture medium containing 10% FBS and incubated for 24 h. Following this, the cells were treated with various concentrations of the test compounds in 100 μL of 10% FBS culture medium for 48 h. Cell viability was then assessed using the CCK8 kit. In accordance with the manufacturer’s instructions for the reagent, the optical density (OD) at 450 nm was measured using the Multimode Mithras LB 943 microplate reader.

### 4.9. Target Screening of Compound and Diseases

Canonical SMILES information for compounds was retrieved from the CAS SciFinder database “http://www.cas.org/ (accessed on 10 December 2024)”. Subsequently, the retrieved data were imported into the SwissTargetPrediction platform “http://swisstargetprediction.ch/ (accessed on 10 December 2024)” for comprehensive potential target screening. Disease targets were identified using GeneCards databases “https://www.genecards.org/ (accessed on 10 December 2024)”. The key terms “Neurodegenerative diseases”, “Oxidative phosphorylation”, “Mitochondrial potential”, “Mitochondrial biogenesis”, and “Ferroptosis” were applied to the search, and the overlap was identified to determine the neurodegenerative disease targets associated with inflammation and ferroptosis. Furthermore, the compound potential targets were used to search for and identify intersections with previously identified targets to identify the associated targets for compound and neurodegenerative disease.

### 4.10. Core Target–Ingredient Network and Hub Genes

Using online bioinformatics software “https://www.bioinformatics.com.cn (accessed on 10 December 2024)”, the predicted targets of compound were mapped with the disease targets, which were visualized using Venn diagrams. Subsequently, using the STRING platform “https://www.string-db.org (accessed on 10 December 2024)”, specifying “Homo sapiens” and applying a medium confidence threshold for interactions, we created a protein–protein interaction (PPI) network diagram. Subsequently, we used cytoHubba [85,86], a plugin for Cytoscape software (version 3.9.1) [87,88], to identify hub genes. In cytoHubba, we selected top 7 nodes from each of the 12 algorithms, and the genes with degree < 10 were ruled out.

### 4.11. Molecular Docking

The structure of the protein (TLR4: 3FXI) was downloaded from the Protein Data Bank (PDB). The 3D structure of the small molecule was constructed from yinfotek computing platform “https://cloud.yinfotek.com (accessed on 10 December 2024)” and energy minimization was carried out under the MMFF94 force field. AutoDock Vina 1.1.21 software was adopted for molecular docking and PyMol 2.4.0 (The Scripps Research Institute, La Jolla, CA, USA) was used to remove water molecules, salt ions, and small molecules from the protein. The docking box was then set up to encase the entire protein structure. The Lamarckian Genetic Algorithm (LGA) was employed to conduct a conformational search. For docking, all parameters were kept at default. The docking scores are reported as kcal/mol; the more negative the number is, the better the binding. The output docked conformation of the two proteins was considered as the definitive conformation. Discovery Studio Version 4.5 software (Accelrys Software Inc., San Diego, CA, USA) was utilized to analyze the interaction forces, and finally, PyMol 2.4.0 (DeLano Scientific LLC, South San Francisco, CA, USA) was used for visualization.

### 4.12. Cellular Thermal Shift Assay (CETSA)

BV2 cells were lysed with RIPA buffer on ice for 30 min. Following lysis, cells were collected and subjected to five cycles of freeze–thaw. After centrifugation, the supernatants were treated with isobisvertinol (100 μM) or DMSO for 1 h, then transferred to tubes. Samples were exposed to various temperatures for 5 min [89]. Once cooled to room temperature, the samples were centrifuged again, and the resulting supernatants were subjected to Western blot analysis.

### 4.13. Protein Sample Preparation and Western Blotting

Cells (1.5 × 10^5^ cells/well) were seeded in a 24-well plate. After 24 h, cells were treated with fresh medium containing the tested compounds for 1 h, followed by exposure to LPS (1 µg/mL). Control groups were treated with a 0.1% DMSO solution. After 12 h, cells were collected and lysed with cold RIPA. The protein was separated by 10% SDS-PAGE and transferred to the PVDF membrane. Then, the membrane was sealed with 5% skimmed milk at room temperature for 1 h. Membranes coated with transferred protein were treated with primary antibodies to cell proteins at 4 °C overnight. After washing, the membrane and specific species of horseradish peroxidase coupled with the second antibody at 37 °C was incubated for 1 h, and the generated antigen–antibody complex was displayed with the enhanced chemiluminescence kit. We then used the chemiluminescence and fluorescence imaging system to scan the image (Tanon 4600, Tanon, China), and used ImageJ software 1.46r to quantify the band with *β*-actin as the internal parameter.

### 4.14. In Vitro Lipid Peroxidation Analysis

HT22 cells (1.5 × 10^5^ cells/well) were cultured in 6-well plates with 200 μL of DMEM containing 10% FBS and incubated for 24 h. The cells were then treated with RSL3 along with the tested compounds for 3 h. Following the manufacturer’s instructions for BODIPY581/591C11, the cells were stained and incubated at 37 °C for 30 min. After washing with HBSS, fluorescence was measured using a digital microscope (Axio Imager, ZEISS, Oberkochen, Germany).

### 4.15. Mitochondrial Membrane Potential (JC-10)

HT22 cells (1.5 × 10^5^ cells/well) were cultured in 6-well plates with 200 μL of DMEM containing 10% FBS and incubated for 24 h. The cells were then treated with RSL3 along with the tested compounds. After 3 h, the culture medium was aspirated, and 1 mL of JC-10 staining working solution was added. The incubation continued at 37 °C for 20 min in a cell culture incubator. Post-incubation, the cells were washed twice with a staining buffer to remove any residual dye. Subsequently, fresh culture medium was added, and the samples were assessed for mitochondrial membrane potential using a fluorescence microscope to capture the images.

### 4.16. Metabolomics Sample Preparation

Cells were extracted using 1 mL of a solvent mixture containing methanol and water in a 4:1 volume ratio. Samples were vortexed and sonicated for 30 s, followed by three cycles of freeze-thaw to disrupt the cells. The mixture was then stored at −20 °C for 1 h to precipitate proteins. Subsequently, the samples were centrifuged at 4 °C for 15 min at 13,800× *g*. The supernatant was transferred to a new centrifuge tube and dried in a vacuum concentrator. The dried extract was reconstructed with 120 μL of a solvent mixture consisting of acetonitrile and water in a 1:1 volume ratio. After centrifugation at 4 °C for 15 min at 13,800× *g*, 80 μL of the supernatant from each sample was collected for UHPLC-QTOF-MS analysis.

### 4.17. UHPLC-QTOF-MS Analysis

UHPLC-QTOF-MS analysis was performed using an LC system coupled with a Triple TOF 5600+ mass spectrometer (AB SCIEX). Chromatographic separation was achieved on a BEH Amide column (1.7 μm, 2.1 × 100 mm; Waters) and maintained at 40 °C. Mobile phase A consisted of 25 mM NH4OAc and 20 mM NH_4_OH in water (pH 9.15), while mobile phase B was acetonitrile. Elution was carried out using the following gradient at a flow rate of 0.4 mL/min: 0 min, 95% B; 3 min, 75% B; 6 min, 70% B; 7 min, 65% B; 10 min, 40% B; 10.1 min, 95% B; 15 min, 95% B. The mass spectrometer was operated in negative ionization mode under the following source conditions: curtain gas 35 psi, gas 1 60 psi, gas 2 60 psi, source temperature 650 °C, ion spray voltage −4000 V. Information-dependent acquisition (IDA) mode was employed with 10 precursors selected per cycle for fragmentation at collision energy (CE) of 35 ± 15 V and an accumulation time of 50 ms.

### 4.18. UHPLC-Q/TOF MS Data Processing and Statistical Analysis

The MS data files were converted to the mzML format using Pro-teoWizard (version 3), and R (version 4.3.1) software was utilized for peak alignment, retention time (RT) correction, and peak area extraction. Putative metabolites were identified by matching MS/MS fragmentation patterns in the human metabolome database (HMDB) using a secondary scoring system, with the primary MS analysis set to a mass error threshold below 10 ppm. The resulting data were subjected to partial least squares discriminant analysis (PLS-DA) using the “R” package mixOmics.

### 4.19. Construction of Gene-Metabolite Interaction Network

To focus on neuroinflammation and ferroptosis, a comprehensive biochemical pathway analysis of differential metabolites was conducted using an R package against the Smpdb database. The MetScape [90], a cytoscape plugin, was utilized to visualize the metabolite reaction network. Subsequently, R packages were employed for a differential signaling pathway enrichment analysis of relevant genes involved in metabolites, referencing the KEGG and REACTOME databases. Finally, a gene-metabolite interaction network was constructed to elucidate potential gene–metabolite–target interactions encompassing intersecting genes, metabolites, and targets, which were visualized using cytoscape.

### 4.20. Statistical Analysis

Data were expressed as mean ± standard error of the mean and were analyzed by one-way ANOVA (Turkey test) usingPrism 8.0 (GraphPad, La Jolla, CA, USA). Statistical significance was set at * *p* < 0.05, ** *p* < 0.01, and *** *p* < 0.001.

## Data Availability

The raw data supporting the conclusions of this article will be made available by the authors on request.

## Supplementary Materials

The following supporting information can be downloaded at: https://www.mdpi.com/article/10.3390/md23010049/s1, Scheme S1: Isolation procedure for 44 compounds from *Penicillium griseofulvum* MCCC3A00181, Figure S1: Anti-inflammatory effect in BV2 cells of the fermentation extract of *Penicillium griseofulvum* MCCC3A00181, and physicochemical and spectroscopic data of **1**–**44**.
