# Supplementary material for: Deep-Sea-Derived Isobisvertinol Targets TLR4 to Exhibit Neuroprotective Activity via Anti-Inflammatory and Ferroptosis-Inhibitory Effects
Source: Mar Drugs. 2025 Jan 20;23(1):49. doi: 10.3390/md23010049 (PMC11766622; doi:10.3390/md23010049)
Supplement: Supplementary file 1 [file marinedrugs-23-00049-s001.zip › marinedrugs-3411053-supplementary.pdf]

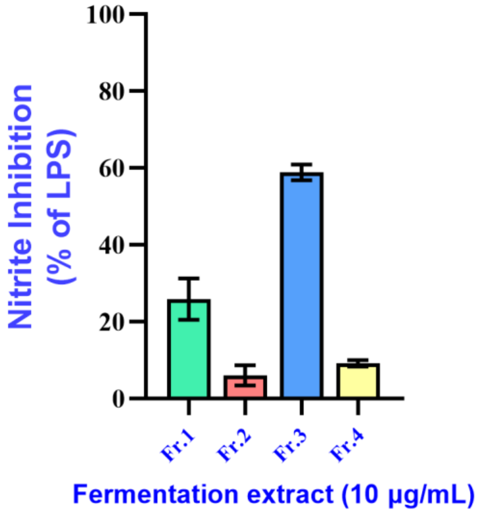

Figure S1. Anti-inflammatory effect in BV2 cells of the fermentation extract of *Penicillium griseofulvum* MCCC 3A00181.

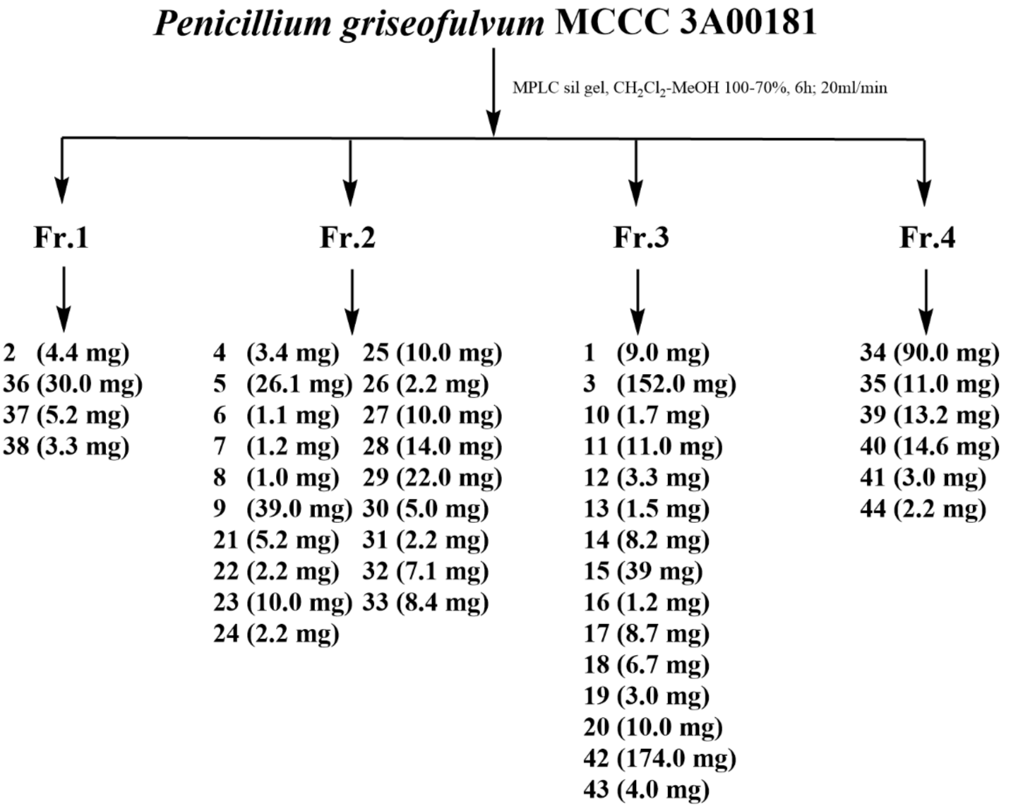

Scheme S1. Isolation procedure for 44 compounds from *Penicillium griseofulvum* MCCC 3A00181.

Physicochemical and Spectroscopic Data of 1–44:

22*E*-5*α*,8*α*-Epidioxyergosta-6,9(11),22-trien-3*β*-ol (**1**): $C_{28}H_{44}O_3$, white powder; [*α*]25 D +42.2 (c 0.80, MeOH); ^{1}H-NMR (400 MHz, $CDCl_3$), $δ_H$: 0.83 (3H, d, *J* = 6.8 Hz), 0.85 (3H, d, *J* = 6.5 Hz), 0.86 (3H, s), 0.92 (3H, s), 0.93 (3H, d, *J* = 6.8 Hz), 1.02 (3H, d, *J* = 6.6 Hz), 5.15 (1H, dd, *J* = 15.2, 8.4 Hz), 5.24 (1H, dd, *J* = 15.2, 8.4 Hz), 6.27 (1H, d, *J* = 8.5 Hz), 6.53 (1H, d, *J* = 8.5 Hz); ^{13}C-NMR (100 MHz, $CDCl_3$), $δ_C$: 12.2 q, 17.0 q, 17.5 q, 19.0 q, 19.3 q, 20.3 q, 20.3 t, 23.1 t, 28.4 t, 29.5 t, 33.0 d, 34.6 t, 36.4 t, 36.8 s, 39.3 t, 39.7 d, 42.9 d, 44.4 s, 51.2 d, 51.7 d, 56.2 d, 65.6 d, 79.5 s, 82.3 s, 130.4 d, 132.1 d, 135.3 d, 135.5 d; HRESIMS *m*/*z* 429.6640 $[M+H]^+$ (calc. for $C_{28}H_{45}O_3$, 429.6645).

22*E*-7*α*-Methoxy-5*α*,6*α*-epoxyergosta-8(14),22-dien-3*β*-ol (**2**): $C_{29}H_{46}O_3$, white powder; [*α*]25 D -35.9 (*c* 0.44, MeOH); ^{1}H-NMR (400 MHz, $CDCl_3$), $δ_H$: 0.82 (3H, d, *J* = 6.4 Hz), 0.85 (3H, d, *J* = 6.4 Hz), 0.86 (3H, s), 0.88 (3H, s), 0.94 (3H, d, *J* = 6.8 Hz), 1.03 (3H, d, *J* = 6.6 Hz), 3.22 (1H, d, *J* = 3.1 Hz), 3.41 (1H, s), 3.93 (1H, m), 4.18 (1H, s), 5.20 (1H, m), 5.21 (1H, m); ^{13}C-NMR (100 MHz, $CDCl_3$), $δ_C$: 16.5 q, 17.6 q, 18.2 q, 19.2 t, 19.6 q, 19.9 q, 21.2 q, 24.9 t, 27.2 t, 31.1 t, 32.1 t, 33.1 d, 35.9 s, 36.5 t, 39.3 d, 39.6 t, 40.2 d, 42.8 d, 43.1 s, 54.5 q, 56.7 d, 58.5 d, 65.2 s, 68.8 d, 72.6 d, 122.5 s, 132.1 d, 135.3 d, 153.3 s; HRESIMS *m*/*z* 465.3318 $[M+Na]^+$ (calc. for $C_{29}H_{46}O_3Na$, 465.3345).

Threo-23-*O*-methylneocyclocitrinol (**3**): white powder; [*α*]25 D +43.3 (*c* 0.15, MeOH); ^{1}H-NMR (400 MHz, CD_3OD), $δ_H$: 0.65 (3H, s), 1.07 (3H, d, *J* = 6.5 Hz), 1.79 (3H, s), 3.62 (1H, qd, *J* = 6.5, 6.3 Hz), 3.80 (1H, dd, *J* = 8.6, 6.3 Hz), 5.29 (1H, d, *J* = 8.0 Hz), 5.51 (1H, d, *J* = 8.2 Hz), 5.53 (1H, s); ^{13}C-NMR (100 MHz, CD_3OD), $δ_C$: 17.4 q, 18.9 q, 22.8 t, 24.2 t, 27.6 t, 27.8 t, 35.7 t, 38.0 t, 41.6 t, 46.9 s, 48.6 d, 54.1 d, 54.2 d, 55.3 q, 59.3 d, 64.5 d, 70.3 d, 81.0 d, 122.2 d, 124.2 d, 124.8 d, 13.7 q, 139.5 s, 145.7 s, 157.7 s, 205.4 s; HRESIMS *m*/*z* 437.2668 $[M+Na]^+$ (calc. for $C_{26}H_{38}O_4Na$, 437.2668).

22-Acetylisocyclocitrinol A (**4**): white powder; [*α*]20 D +128.0 (*c* 0.15, $CHCl_3$); ^{1}H-NMR (400 MHz, $CDCl_3$), $δ_H$: 0.86 (3H, s), 1.32 (3H, s), 1.75 (3H, dd, *J* = 6.5, 1.6 Hz), 2.10 (3H, s), 2.78 (1H, dd, *J* = 12.4, 5.7 Hz), 5.08 (1H, d, *J* = 7.5 Hz), 5.45 (1H, m), 5.57 (1H, m), 5.59 (1H, m), 5.81 (1H, m); ^{13}C-NMR (100 MHz, $CDCl_3$), $δ_C$: 14.1 q, 18.1 q, 21.4 q, 21.7 t, 22.7 t, 23.1 q, 27.6 t, 27.6 t, 28.4 t, 35.7 t, 39.4 t, 41.7 t, 46.3 s, 48.6 d, 54.0 d, 55.0 d, 56.0 d, 64.5 d, 74.9 s, 79.6 d, 122.0 d, 125.1 d, 125.2 d, 132.8 d, 145.9 s, 157.1 s, 170.1 s, 205.2 s; HRESIMS *m*/*z* 465.2622 $[M+Na]^+$ (calc. for $C_{27}H_{38}O_5Na$, 465.2617).

Isocyclocitrinol A (**5**): white powder; [*α*]20 D +136.0 (*c* 0.15, $CHCl_3$); ^{1}H-NMR (400 MHz, CD_3OD), $δ_H$: 0.87 (3H, s), 1.21 (3H, s), 1.72 (3H, d, *J* = 6.5 Hz), 3.81 (1H, d, *J* = 8.2 Hz), 5.45 (1H, m), 5.53 (1H, s), 5.57 (1H, m), 5.58 (1H, m); ^{13}C-NMR (100 MHz, CD_3OD), $δ_C$: 14.5 q, 18.2 q, 20.8 t, 22.2 t, 23.9 q, 28.4 t, 28.9 t, 36.8 t, 40.7 t, 42.1 t, 42.3 s, 48.5 d, 55.4 d, 56.8 d, 57.0 d, 65.3 d, 77.7 s, 78.9 d, 123.1 d, 125.7 d, 129.9 d, 131.5 d, 147.6 s, 160.2 s, 207.8 s; HRESIMS *m*/*z* 423.2516 $[M+Na]^+$ (calc. for $C_{25}H_{36}O_4Na$, 423.2511).

Isocyclocitrinol B (**6**): white powder; [*α*]20 D +104.0 (*c* 0.10, MeOH); ^{1}H-NMR (400 MHz, CD_3OD), δ_H: 0.88 (3H, s), 1.22 (3H, s), 1.71 (3H, d, *J* = 6.5 Hz), 5.45 (1H, m), 5.53 (1H, s), 5.57 (1H, m), 5.58 (1H, m); ^{13}C-NMR (100 MHz, CD_3OD), δ_C: 14.5 q, 20.8 t, 22.2 q, 28.5 t, 28.9 t, 36.8 t, 40.7 t, 42.2 s, 47.7 d, 50.0 d, 55.4 d, 56.8 d, 57.0 d, 65.3 d, 77.7 s, 78.9 d, 123.1 d, 125.7 d, 129.9 d, 131.5 d, 147.6 s, 160.2 s, 207.8 s; HRESIMS *m/z* 423.2510 $[M+Na]^+$ (calc. for $C_{25}H_{36}O_4Na$, 423.2511).

Quinolactacin A1 (**7**): white powder; ^{1}H-NMR (400 MHz, CD_3OD), δ_H: 0.69 (3H, t, *J* = 7.4 Hz), 0.84 (1H, m), 0.94 (1H, m), 1.22 (3H, d, *J* = 6.9 Hz), 2.78 (1H, m), 3.88 (3H, s), 4.85 (1H, m), 7.38 (1H, m), 7.72 (2H, m), 8.22 (1H, d, *J* = 7.9 Hz); ^{13}C-NMR (100 MHz, CD_3OD), δ_C: 11.9 q, 12.1 q, 22.4 t, 37.1 q, 37.8 d, 61.7 d, 111.5 s, 117.9 d, 126.0 d, 127.2 d, 129.0 s, 134.3 d, 142.8 s, 165.9 s, 171.6 s, 174.8 s; HRESIMS *m/z* 293.1266 $[M+Na]^+$ (calc. for $C_{16}H_{18}N_2O_2Na$, 293.1266).

Quinolactacin A2 (**8**): white powder; ^{1}H-NMR (400 MHz, CD_3OD), δ_H: 0.52 (3H, d, *J* = 6.9 Hz), 1.11 (3H, t, *J* = 7.4 Hz), 1.50 (1H, m), 1.65 (1H, m), 2.28 (1H, m), 3.89 (3H, s), 4.97 (1H, m), 7.43 (1H, m), 7.77 (2H, m), 8.28 (1H, d, *J* = 7.9 Hz); ^{13}C-NMR (100 MHz, CD_3OD), δ_C: 12.0 q, 12.4 q, 21.1 t, 37.0 d, 37.8 q, 59.5 d, 111.5 s, 117.9 d, 126.1 d, 127.2 d, 129.1 s, 134.3 d, 143.0 s, 166.4 s, 171.8 s, 174.9 s; HRESIMS *m/z* 293.1267 $[M+Na]^+$ (calc. for $C_{16}H_{18}N_2O_2Na$, 293.1266).

Quinolactacin-H (**9**): white crystal; ^{1}H-NMR (400 MHz, CD_3OD), δ_H: 0.46 (3H, t, *J* = 6.6 Hz), 0.75 (3H, t, *J* = 7.6 Hz), 3.86 (3H, s), 7.57(1H, d, *J* = 2.0 Hz), 7.72 (1H, d, *J* = 4.8 Hz), 8.23 (1H, t, *J* = 7.2 Hz); ^{13}C-NMR (100 MHz, CD_3OD), δ_C: 11.9 q, 12.0 q, 12.4 q, 18.1 q, 29.0 t, 37.1 d, 37.8 d, 111.4 s, 117.9 d, 126.0 d, 127.0 d, 128.9 s, 134.3 d, 142.7 s, 165.8 s, 171.5 s, 174.7 s; HRESIMS *m/z* 285.1586 $[M+H]^+$ (calc. for $C_{17}H_{21}N_2O_2$, 285.1598).

Pyrrospirone C (**10**): $C_{33}H_{43}NO_5$, colorless amorphous powder; ^{1}H-NMR (600 MHz, $CDCl_3$), δ_H: 0.54 (1H, q, *J* = 11.9 Hz), 0.81 (1H, t, *J* = 12.0 Hz), 0.91 (3H, d, *J* = 6.3 Hz), 1.21 (3H, d, *J* = 6.3 Hz), 1.36 (3H, s), 1.83 (3H, s), 1.91 (3H, s), 3.20 (3H, s), 3.22 (1H, m), 3.40 (1H, m), 4.53 (1H, m), 5.09 (1H, m), 6.75 (1H, dd, *J* = 8.1, 2.5 Hz), 6.79 (1H, dd, *J* = 8.1, 2.5 Hz), 6.83 (1H, dd, *J* = 8.1, 2.2 Hz), 6.98 (1H, dd, *J* = 8.5, 2.2 Hz); ^{13}C-NMR (150 MHz, $CDCl_3$), δ_C: 16.1 q, 19.7 q, 20.9 q, 22.8 q, 26.8 q, 27.2 d, 28.0 d, 35.8 t, 41.8 s, 45.6 t, 45.7 t, 45.9 d, 48.8 t, 49.2 q, 50.2 t, 50.4 s, 50.9 d, 52.3 d, 57.9 s, 61.1 d, 72.3 d, 87.1 d, 92.6 s, 118.6 d, 124.5 d, 126.6 d, 132.7 d, 132.7 s, 133.0 d, 140.6 s, 158.9 s, 175.9 s, 199.6 s; HRESIMS *m/z* 534.3217 $[M+H]^+$ (calcd for $C_{33}H_{44}NO_5$, 534.3219).

GKK1032B (**11**): $C_{33}H_{41}NO_4$, white powder; ^{1}H-NMR (600 MHz, $CDCl_3$), δ_H: 0.66 (1H, q, *J* = 12.7 Hz), 0.82 (1H, t, *J* = 12.1 Hz), 0.93 (3H, d, *J* = 6.2 Hz), 1.03 (1H, m), 1.19 (3H, s), 1.20 (3H, s), 1.20 (3H, s), 1.84 (1H, m), 1.86 (1H, m), 1.92 (3H, s), 1.98 (1H, m), 2.00 (1H, m), 2.22 (1H, m), 2.42 (1H, t, *J* = 11.5 Hz), 2.81 (1H, d, *J* = 5.2 Hz), 3.08 (1H, d, *J* = 9.8 Hz), 3.70 (1H, dd, *J* = 13.0, 8.3 Hz), 3.84 (1H, m), 4.24 (1H, m), 4.68 (1H, d, *J* = 17.6 Hz), 4.77 (1H, d, *J* = 11.0 Hz), 5.10 (1H, m), 5.25 (1H, m), 6.73 (1H, m) , 6.97 (1H, m), 7.05 (1H, m), 7.24 (1H, dd, *J* = 8.3, 1.8 Hz); ^{13}C-NMR (150 MHz, $CDCl_3$), δ_C: 16.4 q, 19.7 q, 20.5 q, 22.8 q, 24.9 q, 27.1 d, 27.9 d, 34.7 t, 41.1 s,

41.8 s, 43.5 d, 45.1 t, 49.4 t, 53.9 d, 55.3 d, 60.3 d, 61.4 d, 61.5 d, 93.0 d, 114.0 t, 120.6 d, 126.7 d, 131.8 d, 132 d, 133.4 d, 134.0 s, 138.5 s, 145.3 d, 159.7 s, 170.2 s, 176.9 s, 200.8 s; HRESIMS *m*/*z* 516.3130 $[M+H]^+$ (calcd for $C_{33}H_{42}NO_4$, 516.3036).

11-*O*-Methyl-oxalicumone A (**12**): $C_{19}H_{20}O_9S$, yellow powder; ^{1}H-NMR (400 MHz, CD_3OD), δ_H: 2.40 (3H, s), 2.88 (1H, dd, *J* = 14.1, 6.9 Hz), 2.97 (1H, dd, *J* = 14.1, 4.3 Hz), 3.07 (1H, dd, *J* = 17.5, 9.0 Hz), 3.42 (1H, dd, *J* = 17.5, 8.1 Hz), 3.75 (3H, s), 3.82 (3H, s), 4.14 (1H, t, *J* = 8.5 Hz), 4.36 (1H, dd, *J* = 6.9, 4.3 Hz), 6.64 (1H, s), 6.85 (1H, s); ^{13}C-NMR (100 MHz, CD_3OD), δ_C: 22.2 q, 36.4 t, 38.8 t, 52.5 q, 52.6 q, 53.6 q, 72.6 d, 81.1 s, 109.1 d, 110.0 s, 113.7 d, 122.5 s, 148.9 s, 158.6 s, 162.0 s, 173.3 s, 174.4 s, 174.5 s, 180.3 s; HRESIMS *m*/*z* 447.0726 $[M+Na]^+$ (calc. for $C_{19}H_{20}O_9SNa$, 447.0726).

Oxalicumone A (**13**): $C_{18}H_{18}O_9S$, yellow powder; ^{1}H-NMR (600 MHz, CD_3OD), δ_H: 2.42 (3H, s), 2.92 (1H, dd, *J* = 14.0, 6.8 Hz), 3.01 (1H, dd, *J* = 14.1, 4.2 Hz), 3.10 (1H, dd, *J* = 17.5, 8.9 Hz), 3.44 (1H, dd, *J* = 17.3, 8.1 Hz), 3.77 (3H, s), 3.84 (3H, s), 4.16 (1H, t, *J* = 8.5 Hz), 4.38 (1H, dd, *J* = 6.5, 4.4 Hz), 6.66 (1H, s), 6.87 (1H, s); ^{13}C-NMR (150 MHz, CD_3OD), δ_C: 22.2 q, 36.4 t, 38.8 t, 52.4 d, 52.6 q, 53.6 q, 72.6 d, 81.1 s, 109.1 d, 110.0 s, 113.6 d, 122.5 s, 148.9 s, 158.6 s, 162.0 s, 173.3 s, 174.4 s, 174.5 s, 180.2 s; HRESIMS *m*/*z* 433.0566 $[M+Na]^+$ (calc. for $C_{18}H_{18}O_9SNa$, 433.0569).

Penicitrinol A (**14**): $C_{23}H_{26}O_5$, yellow powder; ^{1}H-NMR (400 MHz, CD_3OD), δ_H: 1.27 (3H, d, *J* = 6.8 Hz), 1.28 (3H, d, *J* = 6.8 Hz), 1.32 (3H, d, *J* = 6.8 Hz), 1.38 (3H, d, *J* = 6.8 Hz), 2.10 (3H, s), 2.11 (3H, s), 2.97 (1H, m), 3.06 (1H, m), 4.08 (1H, m), 4.46 (1H, m), 5.65 (1H, s), 6.45 (1H, s); ^{13}C-NMR (100 MHz, CD_3OD), δ_C: 11.0 q, 11.4 q, 19.2 q, 20.1 q, 20.8 q, 22.0 q, 37.5 d, 44.2 d, 66.8 d, 79.1 d, 87.6 d, 100.7 d, 105.8 s, 110 s, 115.2 s, 117.2 s, 132.2 s, 133.3 s, 137.9 s, 138.1 s, 146.9 s, 148.0 s, 154.8 s; HRESIMS *m*/*z* 381.1718 $[M-H]^-$ (calc. for $C_{23}H_{25}O_5$, 381.1702).

Penicitrinone A (**15**): $C_{23}H_{24}O_5$, yellow powder; ^{1}H-NMR (400 MHz, CD_3OD), δ_H: 1.33 (6H, d, *J* = 6.8 Hz), 1.36 (3H, d, *J* = 6.8 Hz), 1.40 (3H, d, *J* = 6.8 Hz), 2.11 (3H, s), 2.20 (3H, s), 3.24 (1H, m), 3.33 (1H, m), 4.61 (1H, m), 5.20 (1H, m), 6.29 (1H, s); ^{13}C-NMR (100 MHz, CD_3OD), δ_C: 10.3 q, 11.1 q, 18.4 q, 18.5 q, 20.4 q, 18.6 q, 34.5 d, 44.2 d, 82.1 d, 87.6 d, 99.4 s, 102.0 s, 102.3 d, 116.5 s, 130.8 s, 131.2 s, 135.3 s, 137.2 s, 139.3 s, 147.4 s, 156.5 s, 157.8 s, 183.6 s; HRESIMS *m*/*z* 381.1727 $[M+H]^+$ (calc. for $C_{23}H_{25}O_5$, 381.1702).

Penicitrinone F (**16**): $C_{24}H_{26}O_5$, yellow powder; ^{1}H-NMR (400 MHz, CD_3OD), δ_H: 1.32 (3H, d, *J* = 6.8 Hz), 1.34 (3H, d, *J* = 6.8 Hz), 1.39 (3H, d, *J* = 6.8 Hz), 1.40 (3H, d, *J* = 6.8 Hz), 2.13 (3H, s), 2.14 (3H, s), 2.23 (3H, s), 3.25 (1H, m), 3.28 (1H, m), 4.63 (1H, m), 5.15 (1H, m); ^{13}C-NMR (100 MHz, CD_3OD), δ_C: 8.2 q, 11.2 q, 11.8 q, 18.7 q, 19.4 q, 19.5 q, 21.1 q, 35.9 d, 45.9 d, 84.0 d, 89.3 d, 100.7 s, 103.5 s, 111.2 s, 118.3 s, 130.7 s, 132.9 s, 137.6 s, 139.2 s, 141.7 s, 149.1 s, 156.2 s, 160.0 s, 184.8 s; HRESIMS *m*/*z* 395.1859 $[M+H]^+$ (calc. for $C_{24}H_{26}O_5$, 395.1858).

Isobisvertinol (**17**): $C_{28}H_{34}O_8$, yellow amorphous; [α]25 D -463.8 (*c* 0.18, MeOH); ^{1}H-NMR (400 MHz, CD_3OD), δ_H: 1.06 (3H, s), 1.39 (3H, s), 1.42 (3H, s), 1.72 (3H, s), 5.78 (1H, m), 6.05 (2H,

m), 6.16 (2H, m), 6.40 (2H, m), 7.05 (1H, m), 7.24 (1H, m); ^{13}C-NMR (100 MHz, CD_3OD), δ_C: 6.8 q, 18.7 q, 18.8 q, 19.2 q, 22.3 q, 25.5 s, 35.9 s, 53.5 q, 58.3 s, 73.8 s, 79.6 s, 100.6 s, 103.8 s, 106 s, 110.2 s, 112.2 d, 120.3 d, 130.8 d, 131.1 d, 136.8 d, 138.9 d, 140.1 d, 142.8 s, 164.5 s, 168.6 s, 179.8 s, 191.6 s, 206.3 s; HRESIMS *m/z* 499.2331 $[M+H]^+$ (calc. for $C_{28}H_{35}O_8$, 499.2332).

Trichodimerol (**18**): $C_{28}H_{32}O_8$, white powder; [α]25 D -105.0 (*c* 0.14, MeOH); ^{1}H-NMR (400 MHz, $CDCl_3$), δ_H: 1.44 (6H, s), 1.47 (6H, s), 1.89 (6H, d, *J* =6.5 Hz), 3.00 (2H, s), 3.20 (1H, s), 6.11-6.34 (6H, m), 7.33 (2H, dd, *J* = 14.7, 10.6 Hz); ^{13}C-NMR (100 MHz, $CDCl_3$), δ_C: 18.7 q, 18.9 q, 21.2 q, 57.5 d, 58.8 s, 78.8 s, 102.7 s, 104.1 s, 118.5 d, 130.9 d, 140.4 d, 143.6 d, 175.9 s, 198.0 s; HRESIMS *m/z* 519.1999 $[M+Na]^+$ (calc. for $C_{28}H_{32}O_8Na$, 519.1995).

Epiremisporine B (**19**): $C_{30}H_{24}O_{12}$, yellow amorphous powder; [α]25 D +524.4 (*c* 0.16, MeOH); ^{1}H-NMR (600 MHz, $CDCl_3$), δ_H: 2.34 (3H, s), 2.35 (3H, s), 2.48 (1H, m), 2.64 (1H, m), 2.89 (1H, m), 3.81 (3H, s), 3.85 (3H, s), 3.91 (1H, d, *J* = 7.6 Hz), 5.23 (1H, d, *J* = 9.0 Hz), 6.61 (1H, s), 6.62 (1H, s), 6.69 (1H, s), 6.73 (1H, s), 12.05 (1H, s), 12.36 (1H, s); ^{13}C-NMR (150 MHz, $CDCl_3$), δ_C: 22.2 q, 22.4 q, 26.3 t, 37.1 d, 43.3 d, 48.3 d, 50.8 q, 53.3 q, 88.7 s, 104.9 s, 107.6 d, 108.3 s, 108.4 d, 108.9 s, 112.6 s, 113 d, 113.1 d, 119.2 s, 147.3 s, 147.5 s, 156.1 s, 157.2 s, 160.4 s, 160.7 s, 166.0 s, 167.4 s, 168.6 s, 169.5 s, 179.0 s, 179.7 s; HRESIMS *m/z* 577.1349 $[M+H]^+$ (calc. for $C_{30}H_{25}O_{12}$, 577.1346).

Epiremisporine B1 (**20**): $C_{31}H_{26}O_{12}$, yellow amorphous powder; [α]25 D +531.6 (*c* 0.07, MeOH); ^{1}H-NMR (600 MHz, $CDCl_3$), δ_H: 1.26 (1H, t, *J* = 7.1 Hz), 2.29 (3H, s), 2.38 (3H, s), 2.48 (2H, dd, *J* = 15.9, 6.6 Hz), 2.79 (1H, ddd, *J* = 11.3, 8.4, 6.6 Hz), 3.70 (3H, s), 3.87 (1H, dd, *J* = 9.0, 8.4 Hz), 4.17-4.12 (2H, m), 5.01 (1H, d, *J* = 9.0 Hz), 6.65 (1H, s), 6.72 (1H, s), 6.79 (1H, s), 6.91 (1H, s), 7.74 (1H, s), 12.16 (1H, s), 12.50 (1H, s); ^{13}C-NMR (150 MHz, $CDCl_3$), δ_C: 21.4 q, 21.8 q, 26.3 t, 36.9 d, 42.9 d, 47.3 d, 52.6 q, 61.3 t, 88.2 s, 105.7 s, 107.6 d, 107.8 s, 108.3 d, 108.4 s, 111.9 d, 111.9 s, 112.3 d, 119.3 s, 13.8 q, 147.4 s, 147.5 s, 155.5 s, 156.8 s, 159.5 s, 159.9 s, 168.1 s, 168.9 s, 169.2 s, 170.8 s, 178.8 s, 179.4 s; HRESIMS *m/z* 591.1497 $[M+H]^+$ (calc. for $C_{31}H_{27}O_{12}$, 591.1503).

Emodin (**21**): $C_{15}H_{10}O_5$, yellow powder; ^{1}H-NMR (400 MHz, d_6-DMSO), δ_H: 2.39 (3H ,s), 6.56 (1H, s), 7.08 (1H, s), 7.13 (1H, s), 7.45 (1H, s), 12.00 (1H, s), 12.06 (1H, s); ^{13}C-NMR (100 MHz, d_6-DMSO), δ_C: 21.5 s, 107.9 d, 108.8 s, 108.9 d, 113.3 s, 120.4 d, 124.1 d, 132.8 s, 135.0 s, 148.2 s, 161.4 s, 164.4 s, 165.7 s, 181.3 s, 189.6 s; HRESIMS *m/z* 269.0522 $[M-H]^-$ (calc. for $C_{15}H_9O_5$, 269.0450).

Citreorosein (**22**): $C_{15}H_{10}O_6$, yellow powder; ^{1}H-NMR (600 MHz, CD_3OD), δ_H: 4.71 (2H, s), 6.57 (1H, d, *J* = 1.5 Hz), 7.21 (1H, d, *J* = 1.5 Hz), 7.28 (1H, s), 7.75 (1H, s); ^{13}C-NMR (150 MHz, CD_3OD), δ_C: 64.1 t, 109.1 d, 110.2 s, 110.6 d, 115.9 s, 118.5 d, 122.2 d, 134.9 s, 136.9 s, 153.1 s, 161.9 s, 163.7 s, 166.8 s, 183.2 s, 191.7 s; HRESIMS *m/z* 287.0511 $[M+H]^+$ (calc. for $C_{15}H_{11}O_6$, 287.0477).

Isorhodoptilometrin (**23**): $C_{17}H_{14}O_6$, yellow crystal; ^{1}H-NMR (400 MHz, CD_3OD), δ_H: 1.10 (3H,

d, J = 6.1 Hz), 2.72 (2H, m), 3.88 (1H, m), 6.57 (1H, d, J = 2.0 Hz), 7.09 (1H, d, J = 2.1 Hz), 7.15 (1H, s), 7.51 (1H, s); ^{13}C-NMR (100 MHz, CD_3OD), δ_C: 23.5 q, 45.1 t, 66.6 d, 107.9 d, 108.8 d, 109.0 s, 113.6 s, 121.0 d, 124.8 d, 132.5 s, 135.1 s, 150.1 s, 161.2 s, 164.5 s, 165.6 s, 181.4 s, 189.8 s; HRESIMS *m/z* 337.0790 $[M+Na]^+$ (calc. for $C_{17}H_{14}O_6Na$, 337.0824).

Physcion (**24**): $C_{16}H_{12}O_5$, white powder; [α]25 D +1.4 (*c* 0.42, MeOH); ^{1}H-NMR (400 MHz, d_6-DMSO), δ_H: 2.32 (3H, s), 3.17 (3H, s), 6.46 (1H, d, J = 1.9 Hz), 6.95 (1H, d, J = 1.9 Hz), 6.96 (1H, m), 7.26 (1H, d, J = 0.9 Hz), 11.83 (1H, s), 11.92 (1H, s); ^{13}C-NMR (100 MHz, d_6-DMSO), δ_C: 21.9 q, 49.0 q, 108.2 d, 109.1 s, 109.2 d, 113.5 s, 120.7 d, 124.4 d, 132.9 s, 135.2 s, 148.5 s, 161.8 s, 164.8 s, 166.0 s, 181.3 s, 189.9 s; HRESIMS *m/z* 307.2670 $[M+Na]^+$ (calc. for $C_{16}H_{12}O_5Na$, 307.0685).

Janthinone (**25**): $C_{16}H_{12}O_5$, yellowish needles; ^{1}H-NMR (400 MHz, $CDCl_3$), δ_H: 2.42 (3H, s), 4.01 (3H, s), 6.61 (1H, s), 6.73 (1H, s), 7.30 (1H, t, J = 6.7 Hz), 7.52 (1H, d, J = 5.6 Hz), 7.72 (1H, t, J = 8.2 Hz), 12.14 (1H, s); ^{13}C-NMR (100 MHz, $CDCl_3$), δ_C: 22.6 q, 53.1 q, 106.9 s, 107.4 d, 111.7 d, 117.5 s, 119.4 d, 122.5 d, 133.5 s, 134.8 d, 149.4 s, 155.6 s, 155.9 s, 161.4 s, 169.7 s, 180.4 s; HRESIMS *m/z* 307.0718 $[M+Na]^+$ (calc. for $C_{16}H_{12}O_5Na$, 307.0685).

Conioxanthone A (**26**): $C_{16}H_{12}O_7$, white powder; ^{1}H-NMR (400 MHz, CD_3OD), δ_H: 3.86 (3H, s), 4.58 (2H, s), 6.75 (1H, s), 6.85 (1H, d, J = 2.1 Hz), 6.95 (1H, d, J = 2.1 Hz), 6.97 (1H, s); ^{13}C-NMR (100 MHz, CD_3OD), δ_C: 54.9 q, 64.5 t, 104.5 d, 105.4 d, 108.4 d, 109.1 s, 111.3 s, 114.0 d, 136.8 s, 153.9 s, 157.5 s, 159.8 s, 162.9 s, 165.7 s, 171.4 s, 181.2 s; HRESIMS *m/z* 339.0481 $[M+Na]^+$ (calc. for $C_{16}H_{12}O_7Na$, 339.0583).

α-Diversonolic ester (**27**): $C_{16}H_{16}O_7$, white solid, [α]25 D -476.0 (*c* 0.20, MeOH); ^{1}H-NMR (400 MHz, d_6-DMSO), δ_H: 2.27 (2H, m), 2.37 (3H, s), 2.51 (1H, s), 2.86 (1H, m), 3.60 (3H, s), 4.13 (1H, m), 6.63 (1H, s), 6.85 (1H, s), 12.31 (1H, s); ^{13}C-NMR (100 MHz, d_6-DMSO), δ_C: 21.9 q, 24.2 t, 25.1 t, 51.9 q, 71.2 d, 74.4 s, 107.3 d, 107.7 s, 111.6 d, 117.3 s, 147.5 s, 155.3 s, 159.5 s, 167.4 s, 173.0 s, 181.0 s; HRESIMS *m/z* 345.0972 $[M+Na]^+$ (calc. for $C_{16}H_{16}O_7Na$, 345.1053).

β-Diversonolic ester (**28**): $C_{16}H_{16}O_7$, white solid, [α]25 D +34.0 (*c* 0.20, MeOH); ^{1}H-NMR (400 MHz, d_6-DMSO), δ_H: 1.96 (2H, m), 2.37 (3H, s), 2.51 (1H, s), 2.86 (1H, m), 3.60 (3H, s), 4.13 (1H, m), 6.63 (1H, s), 6.85 (1H, s), 12.31 (1H, s); ^{13}C-NMR (100 MHz, d_6-DMSO), δ_C: 21.9 q, 24.2 t, 25.1 t, 51.9 q, 71.2 d, 74.4 s, 107.3 d, 107.7 s, 111.6 d, 117.3 s, 147.5 s, 155.3 s, 159.5 s, 167.4 s, 173.0 s, 181.0 s; HRESIMS *m/z* 345.0972 $[M+Na]^+$ (calc. for $C_{16}H_{16}O_7Na$, 345.1053).

Coniochaetone J (**29**): $C_{14}H_{14}O_5$, pale yellow powder; [α]25 D -7.0 (*c* 0.37, MeOH); ^{1}H-NMR (400 MHz, CD_3OD), δ_H: 2.13 (1H, m), 2.37 (1H, m), 2.86 (1H, m), 3.17 (1H, m), 3.47 (3H, s), 4.67 (2H, s), 4.94 (1H, d, J = 6.7 Hz), 6.79 (1H, s), 7.01 (1H, s); ^{13}C-NMR (100 MHz, CD_3OD), δ_C: 27.0 t, 29.4 t, 55.9 q, 62.8 t, 79.5 d, 104.5 d, 108.8 d, 109.3 s, 119.5 s, 150.9 s, 157.6 s, 160.9 s, 175.0 s, 181.1 s; HRESIMS *m/z* 295.0916 $[M+Na]^+$ (calc. for $C_{14}H_{14}O_5Na$, 295.0841).

(–)-Coniochaetone E (**30**): $C_{14}H_{14}O_4$, white powder; [α]25 D -35.0 (*c* 0.10, MeOH); ^{1}H-NMR (400

MHz, d_6-DMSO), δ_H: 1.99 (1H, m), 2.26 (1H, m), 2.37 (3H, s), 2.84 (1H, m), 3.13 (1H, m), 3.32 (3H, s), 4.82 (1H, d, *J* = 6.7 Hz), 6.68 (1H, s), 6.93 (1H, s), 12.63 (1H, s); ^{13}C-NMR (100 MHz, d_6-DMSO), δ_C:21.4 q, 26.7 t, 29.4 t, 56.1 q, 78.6 d, 107.5 s, 107.9 d, 111.9 d, 119.1 s, 146.6 s, 156.5 s, 159.9 s, 174.3 s, 180.2 s; HRESIMS *m*/*z* 269.0784 [M+Na]$^+$ (calc. for $C_{14}H_{14}O_4Na$, 269.0790).

(+)-Coniochaetone E (**31**): $C_{14}H_{14}O_4$, white powder; [α]25 D +35.0 (*c* 0.10, MeOH); ^{1}H-NMR (400 MHz, d_6-DMSO), δ_H: 1.99 (1H, m), 2.26 (1H, m), 2.37 (3H, s), 2.84 (1H, m), 3.13 (1H, m), 3.32 (3H, s), 4.82 (1H, d, *J* = 6.7 Hz), 6.68 (1H, s), 6.93 (1H, s), 12.63 (1H, s); ^{13}C-NMR (100 MHz, d_6-DMSO), δ_C: 21.7 q, 26.9 t, 29.7 t, 56.3 q, 78.8 d, 107.8 s, 107.9 d, 111.9 d, 119.3 s, 147.0 s, 156.8 s, 160.2 s, 174.6 s, 180.5 s; HRESIMS *m*/*z* 269.0776 [M+Na]$^+$ (calc. for $C_{14}H_{14}O_4Na$, 269.0790).

3-(2,6-Dihydroxyphenyl)-4-hydroxy-6-methyl-isobenzofuran-1(3H)-one (**32**): $C_{15}H_{12}O_5$, colorless crystal; [α]25 D -0.6 (*c* 0.7, MeOH); ^{1}H-NMR (400 MHz, CD_3OD), δ_H: 2.14 (3H, s), 6.10 (1H, s), 6.96 (1H, m), 6.96 (1H, s), 7.28 (1H, s), 7.30 (1H, m); ^{13}C-NMR (100 MHz, CD_3OD), δ_C: 21.5 q, 76.7 d, 106.3 s, 108.6 d, 116.2 d, 120.7 d, 130.2 s, 130.8 d, 138.4 s, 141.6 s, 153.6 s, 175.0 s; HRESIMS *m*/*z* 295.0610 [M+Na]$^+$ (calc. for $C_{15}H_{12}O_5Na$, 295.0685).

Bis(2-ethylhexyl) benzene-1,2-dicarboxylate (**33**): $C_{24}H_{38}O_4$, colorless oil; ^{1}H-NMR (400 MHz, CD_3OD), δ_H: 0.92 (6H, m), 0.94 (6H, m), 1.67-1.72 (2H, m), 4.20 (4H, m), 7.72-7.74 (2H, m), 7.54-7.56 (2H, m); ^{13}C-NMR (100 MHz, CD_3OD), δ_C: 11.0 q, 14.0 q, 23.0 t, 23.8 t, 28.9 t, 30.4 t, 38.7 d, 68.2 t, 128.8 d, 130.9 d, 132.5 d, 167.8 s; HRESIMS *m*/*z* 413.2804 [M+Na]$^+$ (calc. for $C_{24}H_{38}O_4Na$, 413.2772).

Sclerotinin C (**34**): $C_{10}H_{12}O_4$, brown oil, [α]25 D -20.9 (*c* 0.45, MeOH); ^{1}H-NMR (400 MHz, CD_3OD), δ_H: 1.12 (6H, d, *J* = 6.8 Hz), 2.07 (3H, s), 3.05 (1H, m), 3.88 (1H, m), 6.25 (1H, s); ^{13}C-NMR (100 MHz, CD_3OD), δ_C:10.6 q, 16.4 q, 19.8 q, 43.2 d, 71.8 d, 102.2 s, 104.7 d, 114.2 s, 149.9 s, 160.0 s, 160.5 s, 178.3 s; HRESIMS *m*/*z* 223.0926 [M+Na]$^+$ (calc. for $C_{10}H_{13}O_4$, 223.0892).

Stoloniferol B (**35**): $C_{12}H_{14}O_4$, colorless needles, [α]25 D -114.0 (*c* 0.50, MeOH); ^{1}H-NMR (400 MHz, CD_3OD), δ_H: 1.31 (3H, d, *J* = 7.1 Hz), 1.34 (3H, d, *J* = 6.6 Hz), 2.10 (3H, s), 2.99 (1H, q, *J* = 7.0 Hz), 4.70 (1H, q, *J* = 6.5 Hz), 6.36 (1H, s), 11.34 (1H, s); ^{13}C-NMR (100 MHz, CD_3OD) δ_C: 9.9 q, 19.6 q, 19.9 q, 34.7 d, 80.1 d, 99.8 s, 101.3 d, 113.8 s, 142.9 s, 161.7 s, 162.2 s, 168.8 s; HRESIMS *m*/*z* 221.0810 [M-H]$^-$ (calc. for $C_{12}H_{13}O_4$, 221.0814).

Citrinin (**36**): $C_{13}H_{14}O_5$, yellowish white powder, [α]25 D -21.0 (*c* 0.30, MeOH); ^{1}H-NMR (400 MHz, CD_3OD), δ_H: 1.18 (3H, d, *J* = 7.1 Hz), 1.30 (3H, d, *J* = 6.6 Hz), 1.97 (3H, d, *J* = 2.2 Hz), 2.98 (1H, q, *J* = 7.2 Hz), 4.78 (1H, q, *J* = 6.7 Hz), 8.23 (1H, s), 15.10 (1H, s), 15.92 (1H, s); ^{13}C-NMR (100 MHz, CD_3OD), δ_C: 9.2 q, 18.0 q, 18.3 q, 34.3 d, 81.7 d, 100.0 s, 107.1 s, 122.7 s, 139.3 s, 163.2 d, 174.4 s, 177.0 s, 183.5 s; HRESIMS *m*/*z* 273.0875 [M+Na]$^+$ (calc. for $C_{13}H_{14}O_5Na$, 273.0841).

Decarboxydihydrocitrinin (**37**): $C_{12}H_{16}O_3$, colorless amorphous powder, [α]25 D +367.7 (*c* 0.22, MeOH); ^{1}H-NMR (400 MHz, CD_3OD), δ_H: 1.20 (6H, d, *J* = 6.6 Hz), 2.03 (3H, s), 2.62 (1H, m),

3.88 (1H, m), 4.60 (1H, m), 6.22 (1H, s); ^{13}C-NMR (100 MHz, CD_3OD), δ_C: 10.3 q, 18.2 q, 20.8 q, 36.4 d, 60.3 t, 75.8 d, 100.8 d, 112.3 s, 114.2 s, 138.5 s, 152.0 s, 155.4 s; HRESIMS *m/z* 209.1091 $[M+H]^+$ (calc. for $C_{12}H_{17}O_3Na$, 209.1099).

Guhypoxylonol C (**38**): $C_{13}H_{18}O_3$, colorless powder, $[\alpha]25$ D -11.5 (*c* 0.33, MeOH); ^{1}H-NMR (400 MHz, CD_3OD), δ_H: 1.18 (3H, d, *J* = 5.4 Hz), 1.19 (3H, d, *J* = 5.1 Hz), 2.07 (3H, d, *J* = 5.4 Hz), 2.62 (1H, qd, *J* = 7.2, 2.2 Hz), 3.87 (1H, qd, *J* = 6.5, 2.4 Hz), 4.62 (2H, q, *J* = 15.2 Hz); ^{13}C-NMR (100 MHz, CD_3OD), δ_C: 7.7 q, 9.7 q, 16.8 q, 19.6 q, 34.9 d, 59.4 t, 74.6 d, 109.9 s, 113.0 s, 114.5 s, 133.4 s, 148.1 s, 151.9 s; HRESIMS *m/z* 223.1332 $[M+H]^+$ (calc. for $C_{13}H_{19}O_3$, 223.1334).

Phenol A (**39**): $C_{11}H_{16}O_3$, pale-yellow solid, $[\alpha]25$ D -30.0 (*c* 0.13, MeOH); ^{1}H-NMR (400 MHz, CD_3OD), δ_H (ppm): 1.13 (3H, d, *J* = 6.4 Hz), 1.16 (3H, d, *J* = 7.1 Hz), 2.10 (3H, s), 3.08 (1H, m), 3.88 (1H, m), 6.18 (1H, d, *J* = 2.2 Hz), 6.29 (1H, d, *J* = 2.3 Hz); ^{13}C-NMR (100 MHz, CD_3OD), δ_C (ppm): 9.4 q, 15.0 q, 18.1 q, 41.7 d, 70.6 d, 99.8 d, 104.5 d, 113.9 s, 144.3 s, 155.0 s, 155.6 s; HRESIMS *m/z* 197.1174 $[M+H]^+$ (calc. for $C_{11}H_{17}O_3$, 197.1172).

5-[2*R*-2-Hydroxypropane-1-yl]-2,6-dimethlbenzene-1,3-diol (**40**): $C_{11}H_{16}O_3$, yellow powder, $[\alpha]25$ D +109.3 (*c* 0.15, MeOH); ^{1}H-NMR (600 MHz, CD_3OD), δ_H: 1.14 (3H, d, *J* = 6.2 Hz), 2.07 (3H, s), 2.12 (3H, s), 2.53 (1H, dd, *J* = 13.4, 7.5 Hz), 2.78 (1H, dd, *J* = 13.3, 3.0 Hz), 3.90 (1H, m); ^{13}C-NMR (150 MHz, CD_3OD), δ_C: 7.6 q, 10.6 q, 21.4 q, 42.9 t, 67.8 d, 109.4 d, 109.4 s, 114.4 s, 134.7 s, 152.7 s, 153.5 s; HRESIMS *m/z* 197.1174 $[M+H]^+$ (calc. for $C_{11}H_{17}O_3$, 197.1172).

3,5-Dimethylorsellinic acid (**41**): $C_{10}H_{12}O_4$, yellowish white powder; ^{1}H-NMR (600 MHz, CD_3OD), δ_H: 2.11 (3H, s), 2.15 (3H, s), 2.50 (3H, s); ^{13}C-NMR (150 MHz, CD_3OD), δ_C: 8.7 q, 12.3 q, 18.9 q, 105.8 s, 108.8 s, 116.4 s, 138.5 s, 159.0 s, 161.5 s, 174.9 s; HRESIMS *m/z* 197.0736 $[M+H]^+$ (calc. for $C_{10}H_{13}O_4$, 197.0736).

1-Glyceryl linoleate (**42**): $C_{21}H_{38}O_4$; white powder; ^{1}H-NMR (400 MHz, CD_3OD), δ_H: 0.89-0.93 (3H, m), 1.30-1.37 (2H, m), 1.62 (2H, t, *J* = 7.4 Hz), 2.03-2.10 (2H, m), 2.35 (2H, m), 2.78 (2H, m), 3.55 (1H, dd, *J* = 5.5, 1.8 Hz), 3.80 (1H, dd, *J* = 5.8, 4.2 Hz), 4.06 (1H, dd, *J* = 11.4, 6.2 Hz), 4.15 (1H, dd, *J* = 11.4, 4.4 Hz), 5.27-5.42 (2H, m); ^{13}C-NMR (100 MHz, CD_3OD), δ_C: 14.1 q, 22.7 t, 24.9 t, 27.2 t, 29.1 t, 29.3 t, 29.5 t, 29.6 t, 29.7 t, 31.8 t, 31.9 t, 34.1 t, 63.4 t, 65.0 t, 70.2 d, 127.9 d, 129.7 d, 130.0 d, 130.2 d, 174.3 s; HRESIMS *m/z* 377.2804 $[M+Na]^+$ (calc. for $C_{21}H_{38}O_4Na$, 377.2770).

Glycerol-1-monooleate (**43**): $C_{21}H_{40}O_4$; white powder; ^{1}H-NMR (400 MHz, CD_3OD), δ_H: 0.90 (3H, t, *J* = 7.0 Hz), 1.28-1.32 (2H, m), 1.65 (2H, m), 2.03 (2H, m), 2.35 (2H, d, *J* = 7.5 Hz), 3.62 (2H, dd, *J* = 11.4, 5.8 Hz), 3.72 (2H, dd, *J* = 11.5, 3.5 Hz), 3.95 (2H, t, *J* = 4.6 Hz), 4.18 (2H, dd, *J* = 11.6, 6.2 Hz), 4.23 (2H, dd, *J* = 11.6, 4.6 Hz), 5.36 (1H, m); ^{13}C-NMR (100 MHz, CD_3OD), δ_C:14.4 q, 23.7 t, 26.0 t, 28.1 t, 30.2 t, 30.2 t, 30.3 t, 30.3 t, 30.5 t, 30.6 t, 30.8 t, 30.8 t, 33.1 t, 34.9 t, 64.1 t, 66.5 t, 71.2 d, 130.8 d, 130.9 d, 175.5 s; HRESIMS *m/z* 379.2960 $[M+Na]^+$ (calc. for $C_{21}H_{40}O_4Na$, 379.1927).

Squalene (**44**): $C_{30}H_{50}$, colorless oil; ^{1}H-NMR (400 MHz, CD_3OD), δ_H: 1.59 (18H, s), 1.66 (6H, s), 1.98 (8H, m), 2.01 (4H, m), 2.07 (8H, m), 5.09 (4H, m), 5.13 (2H, m); ^{13}C-NMR (100 MHz, CD_3OD), δ_C: 16.1 q, 17.7 q, 25.9 q, 27.5 t, 27.8 t, 29.2 t, 40.8 t, 40.9 t, 125.5 d, 125.6 d, 132.0 s, 135.8 s, 135.9 s; HRESIMS *m*/*z* 433.3946 $[M+Na]^+$ (calc. for $C_{30}H_{50}Na$, 433.3913).
